# Pilot study of extended-release lorcaserin for cocaine use disorder among men who have sex with men: A double-blind, placebo-controlled randomized trial

**DOI:** 10.1371/journal.pone.0254724

**Published:** 2021-07-15

**Authors:** Glenn-Milo Santos, Janet Ikeda, Phillip Coffin, John E. Walker, Tim Matheson, Matthew McLaughlin, Jennifer Jain, Eric Vittinghoff, Steven L. Batki

**Affiliations:** 1 Department of Community Health Systems, University of California, San Francisco, CA, United States of America; 2 Center on Substance Use and Health, San Francisco Department of Public Health, San Francisco, CA, United States of America; 3 Division of HIV, Infectious Disease & Global Medicine, University of California, San Francisco, CA, United States of America; 4 Department of Psychiatry and Behavioral Sciences, University of California, San Francisco, CA, United States of America; 5 Department of Epidemiology and Biostatistics, University of California, San Francisco, CA, United States of America; 6 San Francisco VA Health Care System (SFVAHCS), San Francisco, CA, United States of America; Centre for Addiction and Mental Health, CANADA

## Abstract

**Objective:**

To determine if men who have sex with men (MSM) with cocaine use disorder (CUD) and actively-using cocaine could be enrolled and retained in a pharmacologic intervention trial of lorcaserin—a novel 5-HT_2c_R agonist—and determine the degree to which participants would adhere to study procedures.

**Methods:**

This was a phase II randomized, double-blind, placebo-controlled pilot study with 2:1 random parallel group assignment to daily extended-release oral lorcaserin 20 mg versus placebo (clinicaltrials.gov identifier-NCT03192995). Twenty-two of a planned 45 cisgender MSM with CUD were enrolled and had weekly follow-up visits during a 12-week treatment period, with substance use counseling, urine specimen collection, and completion of audio-computer assisted self-interview (ACASI) behavioral risk assessments. Adherence was measured by medication event monitoring systems (MEMS) caps and self-report. This study was terminated early because of an FDA safety alert for lorcaserin’s long-term use.

**Results:**

Eighty-six percent completed the trial, with 82% of weekly study follow-up visits completed. Adherence was 55.3% (lorcaserin 51.6% vs. placebo 66.2%) by MEMS cap and 56.9% (56.5% vs. placebo 57.9%) by self-report and did not differ significantly by treatment assignment. Intention-to-treat analyses (ITT) did not show differences in cocaine positivity by urine screen between the lorcaserin and placebo groups by 12 week follow-up (incidence risk ratio [IRR]: 0.96; 95%CI = 0.24–3.82, P = 0.95). However, self-reported cocaine use in timeline follow-back declined more significantly in the lorcaserin group compared to placebo (IRR: 0.66; 95%CI = 0.49–0.88; P = 0.004).

**Conclusion:**

We found that it is feasible, acceptable, and tolerable to conduct a placebo-controlled pharmacologic trial for MSM with CUD who are actively using cocaine. Lorcaserin was not associated with significant reductions in cocaine use by urine testing, but was associated with significant reductions in self-reported cocaine use. Future research may be needed to continue to explore the potential utility of 5-HT_2c_R agonists.

## Introduction

Cocaine use in the United States (US) is an unrelenting public health problem with serious negative medical, societal, and economic impacts [1–3]. Its use in the US has also increased during the COVID-19 pandemic [4]. In both powder (cocaine hydrochloride) and crack (free base) forms, cocaine is an addictive stimulant; it is estimated that up to one in six cocaine users may develop a moderate to severe use disorder in their lifetime [2, 5, 6]. Cocaine is associated with a range of negative health consequences, including direct cardiac toxicity, myocardial infarctions, and sudden cardiac death [7–11].

Additionally, cocaine use is a major public health issue among men who have sex with men (MSM). National HIV Behavioral Surveillance (NHBS) data indicate that 37% of MSM in the US used cocaine in the past 12 months, which is 15 times more prevalent than the general population [12, 13]. In San Francisco, cocaine use (past 12 months) climbed from 19% in 2003–2005 to 35% in 2014 [14, 15]. This increase corresponded with a significant decrease (test for trend p<0.001) in methamphetamine use among San Francisco MSM (from 22% to 13%) [15, 16].

In addition, cocaine use plays an important role in the HIV epidemic, particularly for MSM, whom disproportionately account for new HIV infections. It is associated with HIV-related risks (e.g., sexual risk behaviors [17–23] and needle-sharing during injection drug use [24, 25]) as well as new HIV infections in multiple longitudinal studies [26–28]. Moreover, among individuals living with HIV, cocaine use has been associated with poor adherence to antiretroviral treatment (ART), HIV disease progression, lower CD4 count, and higher mortality [29–32].

Given the health hazards associated with cocaine use, there is an urgent need to develop effective treatments, particularly for those with cocaine use disorder (CUD) and for populations with higher prevalence of use and associated harm, including MSM. Unlike alcohol and opioid use disorders, there are currently no FDA-approved medications for CUD, which limits treatment options for this pressing public health issue to behavioral interventions alone [33]. Although some positive findings have been associated with certain pharmacologic agents to treat cocaine use, an effective medication for treatment compared to placebo remains elusive in clinical trials [33]. Lorcaserin, a novel selective serotonin (5-HT) receptor agonist that was FDA-approved for weight management, was, at the time of study design, considered to be is a promising agent to treat CUD [34]. Cocaine use increases levels of dopamine (DA), the neurochemical responsible for the reinforcing effects of the drug, and data suggest that DA activity is modulated by the central 5-HT systems [35–37], particular via the 5-HT_2C_ receptor (5-HT_2C_R), which has been identified as the primary 5-HT receptor subtype that inhibits tonic and cocaine-evoked DA activity [37]. 5-HT_2C_R agonists can reduce dopamine release in the nucleus accumbens (NAcc) and frontal neocortex [35, 37]. Preclinical data also support the potential of lorcaserin in reducing cocaine use [36, 38–48]. 5-HT_2C_R selective agonists, including lorcaserin, have been associated with reductions in cocaine self-administration in mice [49, 50] and non-human primates [47, 51]. The only human cocaine trial with lorcaserin was a laboratory study that found that lorcaserin compared to placebo significantly reduced craving for cocaine and delayed cocaine administration [52]. Further support for studying lorcaserin was based on the possibility that lorcaserin had the potential to reduce cocaine use by reducing impulsivity. Excess impulsivity has long been associated with drug use disorders and cocaine use induces greater impulsivity [53]. 5-HT_2C_R agonists have reduced impulsive action [53, 54] and lorcaserin has specifically decreased nicotine-induced impulsive action in animal studies [55]. We hypothesize that lorcaserin’s *dual effects of inhibiting dopamine levels and decreasing impulsivity* may effectively help MSM reduce or stop their cocaine use.

Given the mechanism of action of lorcaserin and the preclinical data supporting its potential for cocaine treatment, we conducted a pilot clinical trial to examine the feasibility, acceptability, and tolerability of lorcaserin among MSM with CUD in an outpatient study. In exploratory analyses, we also examined lorcaserin’s effects on cocaine use and behavioral outcomes including sexual behaviors. This study focused on MSM because the prevalence of CUD is high in this population and effective treatments for CUD can also lead to reductions in HIV transmission among MSM.

## Methods

### Study design and recruitment

This was a randomized, double-blind, placebo-controlled, 12-week parallel group pilot study with 2:1 random assignment to 20 mg of extended-release oral lorcaserin versus placebo. Assignment of two-thirds of participants to the lorcaserin arm was specifically chosen to improve our ability to detect adverse events in the active treatment arm, while preserving our ability to assess recruitment in a study in which randomization to placebo. Participants were recruited via street outreach, recruitment flyers, STD and HIV clinics, needle exchanges, community organizations, MSM bars, online Web sites, and social media. Potential participants completed a brief telephone screen to assess initial eligibility and, if eligible, were scheduled for an in-person screening visit. All participants gave informed consent using UCSF IRB-approved consent forms. A 10-item true/false questionnaire was used to verify participants’ understanding of the trial. The target sample size for the study was 45 participants. With this sample size, we estimated that proportions for our feasibility and acceptability outcomes would be estimated within margins of sampling error (MSEs; i.e., half widths of 95% confidence intervals) of ≤14.4 percentage points, and means with MSEs of 0.30 standard deviations, both typical for a small pilot study. We also estimated that for the sample size of 45, the minimum detectable effects of the intervention on the cocaine use outcomes would need to be substantial, as is typical of a pilot study. In our prior trial for MSM actively using stimulants [56], the within-subject correlation of the repeated measures for urine positivity was 0.45, the urine positivity in the control group was 55%, and the retention was 93% at the end of the study. On this basis, we estimated that the sample would provide 80% power to detect reductions of 29 percentage points in the rate of cocaine urine positivity.

### Early study termination

On February 13, 2020, the Food and Drug Administration (FDA) issued a safety alert after concluding the assessment of the long-term safety of lorcaserin in another clinical trial among 6,000 participants who took lorcaserin for over 4.3 years of treatment and 6,000 who took placebo; all participants were overweight or obese, with cardiovascular disease or multiple cardiovascular disease risk factors (Cardiovascular and Metabolic Effects of Lorcaserin in Overweight and Obese Patients—Thrombolysis in Myocardial Infarction 61 [CAMELLIA-TIMI 61] clinical trial) [57]. The FDA’s analysis of this clinical trial showed an increased risk of cancer, with 7.1% in placebo group being diagnosed with cancer, compared to 7.7% in lorcaserin group. There was no difference in cancer risk noted in the first months of lorcaserin use, but the difference emerged with years of use. In response to the FDA safety alert, active participants in our study at that time (n = 2) were instructed to stop study medication use immediately and all participants (2 active and 20 former) were unblinded of their treatment assignment, informed of the FDA safety alert, and provided a copy of the FDA communication. The FDA’s statement did not recommend special screening for patients who have taken lorcaserin; it recommended that standard screening recommendations for cancer should be implemented for individual patients, regardless of prior lorcaserin treatment. In consultation with the FDA and the study sponsor, our study was paused (i.e., screening and enrollment was stopped), while the FDA determined whether it was feasible or worthwhile to develop revised safety guidelines for investigational studies with lorcaserin for short-term treatment. On May 20, 2020, the FDA determined that it would not be feasible for researchers to acquire the medication lorcaserin, even for short-term investigational use, and no formal guidelines would be developed for studies of lorcaserin with short-term use. At this point, the FDA recommended study termination. The Data and Safety Monitoring Board (DSMB) was advised of the FDA recommendation and the DSMB approved the initiation of study closeout and recommended the study proceed to data analysis based on data collected from 22 participants enrolled at the time of the FDA safety alert. Since termination of the study, a meta-analysis of 4 clinical trials comprising a cumulative sample of 19,771 participants published in December 2020 did not identify a significant increase in risk for cancer with lorcaserin treatment compared to placebo [58].

### Study participants

Participants were eligible if they reported at least one day of active cocaine use during the previous 30 days confirmed by urinalysis or sweat patch at screening (at least one sample positive for cocaine metabolites within two screening and two run-in visits); were seeking treatment for cocaine use and met Diagnostic and Statistical Manual of Mental Disorders (DSM-V) criteria for mild to severe CUD; were HIV negative by rapid antibody test and HIV-pooled RNA test or HIV-positive with medical record of HIV infection and CD4 count; were 18–65 years of age; identified as a cisgender or transgender male who has sex with men; did not have any acute medical or psychiatric illnesses; and had baseline safety labs without clinically significant abnormalities.

We excluded individuals for any psychiatric (e.g., depression with suicidal ideation) or medical condition that would preclude safe study participation or compliance to procedures; any severe current substance use disorder (other than severe cocaine, nicotine, alcohol, or cannabis use disorder) according to DSM-V; testing positive for HIV at screening if previously unaware of HIV infection; a known allergy or previous adverse reaction to lorcaserin; having a current CD4 count < 100 cells/mm3 or HIV viral load greater than 50 copies/mL; moderate to severe liver disease (AST, ALT > 3 times upper limit of normal); severely impaired renal function (creatinine clearance < or = 30 mL/min); use of exclusionary medications that affect the serotonergic neurotransmitter system (e.g., selective serotonin reuptake inhibitors (SSRIs), selective serotonin-norepinephrine reuptake inhibitors (SNRIs), tricyclic antidepressants, or monoamine oxidase inhibitors (MAOIs)); self-reporting a predisposition to priapism; currently participating in other intervention studies or recently participating in the last 30 days; anticipated use of agents that are associated with valvulopathy and/or pulmonary hypertension; currently receiving maintenance treatment with an opioid medication (buprenorphine or methadone); currently being in court-mandated drug treatment; any physical condition affecting drug absorption (e.g., gastrectomy); a 12-lead electrocardiogram (ECG) demonstrating QTc > 450 or a QRS interval > 120 msec at screening; current alcohol use disorder that is judged to require supervised medical detoxification (Clinical Institute Withdrawal Assessment for Alcohol, Revised (CIWA-Ar) score > or = 10); being pregnant; or having a body mass index (BMI) < 15, a BMI ≥ 30 with desire to use weight management medication, or a BMI > 35. The exclusion criteria for BMI ≥ 30 was included because lorcaserin was an FDA-approved treatment for weight loss indicated for obese patients (BMI ≥ 30) and it would have been unethical to enroll individuals who are interested in a weight loss medication because this study was placebo-controlled (i.e., they could have been randomized to placebo). Participants screened with BMI ≥ 30 with desire to use weight management medication were encouraged to speak with their primary health care provider about treatment options for weight loss by study clinicians.

The exclusion criteria for current CD4 count was revised from <200 cells/mm3 to the criteria above of <100 cells/mm3 and approved by the UCSF-IRB on January 29, 2020. The exclusion criteria for CIWA-Ar was added to the protocol after enrollment started, and approved by the UCSF-IRB on February 11, 2019.

### Study procedures

All study procedures were conducted at the San Francisco Department of Public Health (25 Van Ness Avenue, San Francisco, CA). At screening visits, after completing informed consent, participants completed a medical history, vitals, physical, complete blood count, a comprehensive metabolic panel, and a 12-lead ECG. To confirm active cocaine use during screening, the rapid qualitative urine test Medtox Verdict II (Medtox Diagnostics, Burlington, NC) and tamper-evident sweat patches (PharmChek®, PharmChem, Inc., Fort Worth, Tx) were used. Participants who reported being HIV negative or did not know their HIV status received HIV rapid testing (OraQuick HIV 1/2, OraSure Technologies, Inc., Bethlehem, PA) and HIV pooled RNA testing; HIV-positive participants received CD4 count testing. Participants received HIV risk-reduction or "Prevention with Positives" counseling, as appropriate, based on Centers for Disease Control and Prevention (CDC) guidelines [59]. Participants were evaluated for any psychiatric (e.g., depression with suicidal ideation) or medical condition that would preclude safe study participation, as well as any severe current substance use disorder (other than cocaine, nicotine, alcohol, or cannabis use disorder) according to DSM-V. Staff collected extensive participant contact information and two back-up contacts. Eligible participants were scheduled for an enrollment visit.

At enrollment, treatment was assigned using double-blinded block randomization generated using a computer program. The study used randomly selected block sizes of 3 and 6. The study statistician provided the randomization codes to the Safeway compounding pharmacy, which prepared kits corresponding with the treatment assignment (20 mg extended-release lorcaserin or matching placebo) from the randomization code. The study clinicians, staff and participants were all unaware of the treatment assignments for the study drug kits, hence the study was double-blinded. Study clinicians provided training and instructions on dosing of medication during enrollment. Participants were instructed to take one pill each day of extended-release lorcaserin 20mg or placebo. Medications were dispensed in bottles with MEMS caps (AARDEX Group, Liège, Belgium), which are wireless medication monitoring devices that record each opening as a real-time medication event [60]. All participants were asked about potential adverse events at each follow-up visit; symptom-driven physical exams and safety laboratory monitoring were done at weeks 4, 8, and 12. Adverse events were classified using the Division of AIDS (DAIDS) Table for Grading Severity of Adult Adverse Experiences for HIV Prevention Trials Network [61].

Participants were seen every week for substance use counseling and urine tests for cocaine metabolites. Trained staff, supervised by a clinical psychologist, administered brief (20–30 minutes) substance use counseling at follow-up visits, which was modified from a standardized, manual-driven psychosocial substance use counseling program using cognitive behavioral therapy [62] and motivational interviewing techniques [63, 64] incorporating the Stages of Change Model [65]. This platform has been used in brief substance use behavioral interventions and has had high acceptability among MSM who use substances in prior trials [66–69]. Audio-computer assisted self-interviews (ACASI) were used to standardize data collection and minimize reporting bias [70, 71].

HIV risk-reduction counseling and testing were repeated for HIV-negative participants at the final visit. Participants were paid $20 for each screening visit, $10 for run-in visits, $40 for enrollment, $15 for weekly visits, $35 for month 1 and 2 visits, and $50 for the final visit. The study was conducted under the FDA Investigational New Drug Application (IND #134951). Procedures were approved by University of California San Francisco’s Human Research Protection Program (IRB Number 17–21502), and the trial was registered at clinicaltrials.gov (Identifier-NCT03192995).

### Outcome measures

For feasibility outcome measures, we computed the proportions of participants eligible and enrolled among those recruited and screened, the proportion of scheduled visits completed, and the proportion of participants retained to the end of the study to assess feasibility of enrolling and retaining MSM with CUD in a 12-week, randomized, double-blind study of lorcaserin versus placebo. We also tabulated the proportion of participants in each group who correctly guessed their treatment assignment to determine if there was significant evidence of unblinding at the end of the trial.

Acceptability measures were computed from questions on attitudes about trial participation and level of satisfaction with trial procedures. We calculated the proportion of participants who were satisfied/highly satisfied with the study, who would participate in a similar study in the future, and the proportion who would recommend the study to a friend. We evaluated lorcaserin use acceptability by examining the frequency of taking the study drug as measured by the number of MEMS cap openings. Cumulative percent adherence was calculated by dividing the frequency of openings at a given time point divided by the number of days since enrollment. For tolerability measures, we computed the proportion of those experiencing adverse events, both overall and by type, to explore the safety of lorcaserin versus placebo among individuals with CUD who are active users.

To measure cocaine use outcomes at each weekly visit, we used qualitative urine screens and self-reported use in the past week, recognizing the differences in detection windows between urine screens and self-reported use. Prior cocaine treatment studies have also documented greater sensitivity in self-report of cocaine compared to qualitative cocaine urine screens [72, 73]. At each weekly visit, we tested urine samples using rapid qualitative urine test Medtox Verdict II, which are estimated to detect cocaine use from the past 1 to 3 days [74]. Self-reported cocaine use from each day within the prior week was measured using a modified timeline followback [75]. We also calculated the proportion of participants who had any cocaine use in either urine screens or timeline followback to create a composite measure accounting for cocaine use from both urine and self-reported measures.

Standardized measures were used to assess other secondary outcomes, including cocaine craving and severity of cocaine dependence, and sexual risk behavior [18, 75–77]. For monthly visits, the participants completed the measures related to impulsivity, including the Balloon Analog Risk Task (BART) [78] and the Barratt Impulsiveness Scale (BIS) [79].

### Statistical analyses

We used Wilcoxon rank sum and Fisher’s exact tests, as appropriate, to assess the comparability of participants by treatment assignment at baseline, as well as differences in feasibility, acceptability, and tolerability measures. We compared cumulative adherence between treatment groups using the Wilcoxon test.

Using intention to treat (ITT) analyses, we conducted *a priori* exploratory assessments using GEE Poisson models with robust standard errors to account for within-subject correlation, to assess treatment effects on cocaine urine positivity, and self-reported cocaine use using timeline follow-back. The primary ITT analyses excluded cocaine urine test results from week 1, whereas in a sensitivity analysis, we include those results. In additional sensitivity analyses, we included results from missing urine tests as positive for cocaine use, and analyzed treatment effects while adjusting for baseline cocaine use. In all models, the effect of the intervention was estimated by the interaction between treatment assignment and a linear term in time. For GEE diagnostic procedures, departures from linear trends as well as degree to which the within-subject correlation of the outcomes declines over time were evaluated. Although the robust standard errors in our models accounted for declines in within-subject correlation, models with unstructured correlations were also fitted as a sensitivity analysis. Additionally, we examined treatment effects on any cocaine use based on urine positivity or timeline followback. In addition, we conducted as-treated analyses accounting for a time-dependent variable for cumulative medication adherence, calculated from MEMS data, in the analyses examining treatment effects on cocaine urine positivity and self-report; in this analysis, the as-treated effect is estimated by the group-by-adherence interaction. Finally, to evaluate for consecutive weeks of continued abstinence, we compared the “number of beyond-threshold weeks of success” (NOBWOS), defined as the number of consecutive weeks of abstinence lasting through the end of the study [80], using the Wilcoxon test.

We also evaluated treatment effects on cocaine-associated sexual risk behaviors using GEE Poisson, binomial, and negative binomial models using ITT analyses. We used GEE models to explore whether lorcaserin reduced impulsivity as measured by BART and BIS. We also assessed whether lorcaserin reduced cocaine craving and severity of cocaine dependence, using similar methods. Analyses were conducted with STATA 15.0 (StataCorp, College Station, TX).

## Results

### Screening and enrollment

**Fig 1:**
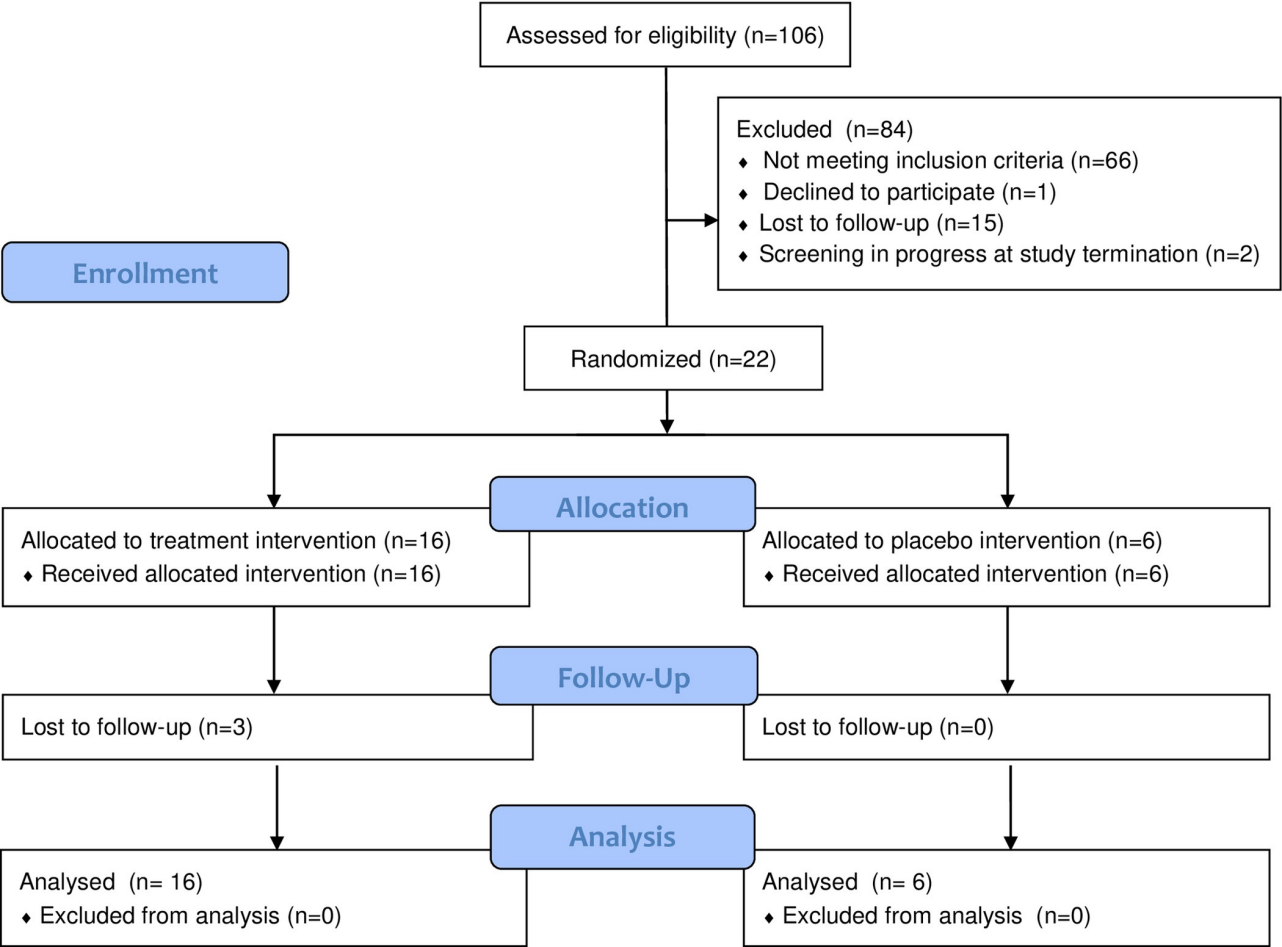
Study participant flow in screening, randomization, follow-up and analysis.

**Fig 1** shows results for screening, recruitment, assignment, and retention for the study period from February 2018 to February 2020. One hundred and six people were assessed for eligibility and consented to participate, of whom 66 were deemed ineligible. The most common reasons for ineligibility were other severe substance use disorders (n = 36); high BMI (n = 8); inability to provide cocaine-positive urine/sweat samples (n = 4); and having ECG values outside the normal range (n = 4). In addition, 15 people were lost to follow-up during screening, 1 declined participation, and 2 were in the screening phase when the study was terminated. At the time of study termination, 22 participants (21% of those screened) were randomized (16 to lorcaserin, 6 to placebo; see **Fig 1**).

### Participant characteristics

We recruited a diverse sample of MSM (41% White, 14% Hispanic/Latino, 23% Black, and 18% Mixed or Other race), of whom 18% were HIV positive (**Table 1**). All participants were cisgender men. No transgender men enrolled in our trial. The median age of participants was 39 (IQR: 32–55). Baseline demographic characteristics were similarly distributed in both groups.

**Table 1:** Baseline demographic and behavioral characteristics of trial participants.

	Control	Treatment	Total
	N = 6	N = 16	N = 22
Age, median (IQR)	40 (39–62)	36 (29–51)	39 (32–55)
Race/Ethnicity			
White	2 (33%)	7 (44%)	9 (41%)
Asian	0 (0%)	1 (6%)	1 (5%)
Black	0 (0%)	5 (31%)	5 (23%)
Latino	2 (33%)	1 (6%)	3 (14%)
Other	2 (33%)	2 (12%)	4 (18%)
Education			
high school or less	1 (17%)	5 (31%)	6 (27%)
some college	3 (50%)	4 (25%)	7 (32%)
college or above	2 (33%)	7 (44%)	9 (41%)
Income			
under $20,000	1 (17%)	7 (44%)	8 (36%)
$20–39,999	3 (50%)	2 (12%)	5 (23%)
$40,000 or more	2 (33%)	7 (44%)	9 (41%)
Employment status			
not employed	1 (17%)	5 (31%)	6 (27%)
full-time	2 (33%)	9 (56%)	11 (50%)
part-time	2 (33%)	2 (12%)	4 (18%)
employed student	1 (17%)	0 (0%)	1 (5%)
Powder cocaine, past 4 weeks			
no use	1 (17%)	3 (19%)	4 (18%)
any use	5 (83%)	13 (81%)	18 (82%)
Crack/Rock Cocaine, past 4 weeks			
no use	4 (67%)	10 (62%)	14 (64%)
any use	2 (33%)	6 (38%)	8 (36%)
Ever hospitalized for cocaine use			
No	5 (83%)	14 (88%)	19 (86%)
Yes	1 (17%)	2 (12%)	3 (14%)
Ever received cocaine treatment			
No	4 (67%)	10 (62%)	14 (64%)
Yes	2 (33%)	6 (38%)	8 (36%)
Craving for Cocaine—Visual Analog Scale	30 (14–45)	40 (2–72)	35 (5–63)
HIV Status			
HIV-positive	0 (0%)	4 (25%)	4 (18%)
HIV-negative	6 (100%)	12 (75%)	18 (82%)
Has regular health care provider			
No	0 (0%)	2 (12%)	2 (9%)
Yes	6 (100%)	14 (88%)	20 (91%)
Depressive Symptoms—Center for Epidemiological Studies Depression (CESD), median (IQR)	10 (7–14)	10 (5–15)	10 (6–14)

### Retention

Overall, the mean percentage of weekly study follow-up visits completed was 82% (SD: 23%). Mean percentage of weekly follow-up visits were similar (P = 0.91) between the lorcaserin (83% [SD: 22]) and the control group (81% [SD: 26]). Overall, 19 participants (86%) were retained at the end of the study, and this study completion rate was similar between groups (lorcaserin 13/16 [81%], placebo 6/6 [100%] P = 0.53).

### Acceptability of procedures

Overall, 74% of participants reported being satisfied/highly satisfied with their study participation. In addition, 89% reported that they would be interested in participating in a similar study in the future, and 89% reported that they would likely recommend participation in the study to a friend. Acceptability of study procedures was similar between the treatment groups.

### Adherence

Overall, the mean percentage of study medication taken by participants as measured by MEMS cap data was 55.3% (SD: 26.6) and similar between groups (lorcaserin 51.6% [SD: 27.7]; placebo 66.2% [SD: 21.7]; P = 0.32). The mean percentage adherence by self-report was 56.9% (SD: 19.3) overall and similar between treatment groups (lorcaserin 56.5% [21.8]; placebo 57.9% [12.2]; P = 0.83).

### Assessment of unblinding

Treatment guessing accuracy between participants in the two groups did not differ significantly (P = 0.63). In the lorcaserin group, 23% guessed they were on lorcaserin. In the placebo group, 50% guessed they were on placebo.

### Tolerability

The overall frequency of adverse events (AEs) was similar between the two groups. There were two serious adverse events (SAEs) in the study that were both deemed unrelated to lorcaserin. One SAE occurred with a participant from the placebo group and involved increased AST (Grade 3 AE) and increased ALT (Grade 2 AE) at month 1 of treatment (resolving to Grade 2 and Grade 1 AEs at the end of study participation). The other SAE was observed in a participant in the lorcaserin group who developed an abscess on his left hip that resolved after a course of antibiotics and was determined to not be related to lorcaserin. The most frequently observed AEs in both groups were hyperglycemia (n = 4; lorcaserin = 3; placebo = 1; P = 0.99), coughing (lorcaserin = 2, placebo = 1; P = 0.8), and abdominal pain (lorcaserin = 2, placebo = 1; P = 0.8). The most frequently observed AEs only present in the lorcaserin group were: rash (n = 3) and diarrhea (n = 3), however, there were no statistically significant differences in the occurrence of these AEs compared to the placebo group (both P = 0.53).

### Exploratory efficacy analyses of cocaine use and secondary outcomes

The proportion of urine-positive samples for the placebo group was 0% (0/6) at baseline, and 20% (1/5) at week 12 visit. In the lorcaserin group, 50% (8/16) of samples were positive at baseline, and 58% (7/12) at week 12 visit. The proportion of urine-positive samples at baseline were not statistically significantly different between groups (P = 0.051). In intention-to-treat analyses (ITT) summarized in **Table 2**, changes in cocaine positivity by urinalyses were similar between lorcaserin and placebo by 12 week follow-up (incidence risk ratio [IRR]: 0.96; 95%CI = 0.24–3.82, P = 0.95), with no evidence of departures from linearity (P = 0.37). The within-subject correlation for urine positivity declined over time. Results were similar for sensitivity analyses including week 1 results, imputing positive results from missing urines, using GEE models with an unstructured correlation, and adjusting for baseline urine-positivity.

**Table 2:** Exploratory efficacy analyses of primary and secondary outcome with sensitivity analyses.

**Primary Outcome:** Cocaine Urine Positivity			Incidence		(95% CI)		P-value
			Risk Ratio (IRR)				
Intention-to-treat analyses	Treatment effect		0.96		(0.24–3.82)		0.954
	Sensitivity Analyses	Inclusion of week 1 results	1.14		(0.16–8.10)		0.899
		Imputing of positive results from missing urines	0.70		(0.24–2.03)		0.507
		Treatment effect using unstructured correlation	2.87		(0.46–17.87)		0.258
		Adjusting for baseline urine positivity	3.11		(0.74–13.11)		0.122
As-treated analyses	Cumulative adherence and treatment effects for urine positivity		1.01		(0.99–1.03)		0.61
**Primary Outcome:** Cocaine Use by self-report in timeline followback			IRR		(95% CI)		P-value
Intention-to-treat analyses	Treatment effect		0.66		(0.49–0.88)		0.004
	Sensitivity analysis	Inclusion of week 1 results	0.66		(0.500.89)		0.005
		Treatment effect using unstructured correlation	0.60		(0.45–0.81)		0.001
		Adjusting for cocaine use week before enrollment	0.68		(0.51–0.91)		0.009
As-treated analyses	Cumulative adherence and treatment effects for self-reported cocaine use		1.01		(0.99–1.02)		0.34
**Primary Outcome:** Any cocaine use based on urine positivity or timeline followback			IRR		(95% CI)		P-value
Intention-to-treat analysis	Treatment effect		0.76		(0.55–1.04)		0.085
**Secondary Outcomes**			Coefficient		(95% CI)		P-value
Cocaine craving—Visual Analog Scale			10.39		(-1.76–22.53)		0.09
Severity of Dependence Scale for Cocaine			0.28		(-3.43–3.99)		0.88
Balloon Analog Risk Task (BART)			-6.03		(-17.1–5.04)		0.29
Barratt Impulsiveness Scale Score			3.40		(-4.50–11.29)		0.40
Depression Score (CES-D)			2.86		(-4.95–10.67)		0.47
**Sexual Behavior Outcomes**			Risk Ratio (RR)		(95% CI)		P-value
Number of male partners			0.03		(0.00–1.24)		0.065
Number of male partners who use cocaine with			0.01		(0.00–6.58)		0.152
Anal intercourse with HIV serodiscordant partners			0.54		(0.17–1.67)		0.283
Condomless anal intercourse with HIV serodiscordant partners			0.05		(0.00–1.93)		0.107
**Other Secondary Outcomes**			Lorcaserin	Placebo		Overall	P-value
MEMS Caps Percent Adherence, mean (SD)			51.64 (27.73)	66.17 (21.70)		55.3 (26.6)	0.77
Self-reported Percent Adherence, mean (SD)			56.51 (21.76)	57.99 (12.18)		56.9 (19.3)	0.83
Number of beyond-threshold weeks (NOBWOS)*, median (IQR)			0 (0–0.5)	1.5 (0–5)		0 (0–1)	0.27

The proportion of past-week cocaine use self-reported from timeline followback for the placebo group was 100% (6/6) at baseline, and 100% (6/6) at week 12 visit. In the lorcaserin group, 75% (12/16) self-reported past-week cocaine use at baseline, and 54% (7/13) at week 12 visit. The proportion of self-reported past-week cocaine use data at baseline were not statistically significantly different between groups (P = 0.541). In ITT analyses, self-reported cocaine use in timeline follow-back declined significantly in the lorcaserin group compared to placebo (IRR: 0.66; 95%CI = 0.49–0.88; P = 0.004), with no evidence of departure from linearity (P = 0.56). The within-subject correlation for urine positivity declined over time. Results were similar and remain statistically significant in favor of lorcaserin in sensitivity analyses including week 1 results, using GEE models with an unstructured correlation, and adjusting for baseline urine-positivity.

In ITT analyses on the composite outcome for any cocaine use based on urine positivity or reported use in timeline followback, results were similar between the lorcaserin and placebo groups (IRR: 0.76; 95% CI = 0.55–1.04; P = 0.085). We did not observe significant treatment effects on the secondary outcomes of impulsivity, depression, craving, sexual risk behaviors, and NOBWOS (see Table 2; all P>0.05). In as-treated analyses accounting for cumulative adherence, cocaine urine positivity (IRR: 1.01; 95% CI = 0.99–1.03; P = 0.61) and self-report (IRR: 1.01; 95% CI = 0.99–1.02; P = 0.34) were also similar between treatment groups.

## Discussion

This study demonstrated that it was feasible to enroll and retain cisgender MSM with CUD who are actively using cocaine in a pharmacologic intervention trial with excellent rates of participation in study visits, procedures, and follow-up evaluations. In addition, our participants reported high acceptability of study procedures and moderate levels of study medication adherence.

Due to its early termination, this study’s final sample is much too small to estimate the treatment effect reliably enough for sample size determination for a larger efficacy trial. Nevertheless, this pilot may provide important information on appropriate modeling techniques given the distribution of the data (e.g., based on the pattern of treatment effect, the intraclass correlation of the repeated measures, and whether and how steeply the within-subject correlation declines over time, and the loss to follow-up rate). In our data, we observed an increasing treatment effect with no evidence of departure from a linearly increasing effect, suggesting that this model was appropriate. Additionally, we observe some decline in the within-subject correlation over time. However, as noted, fitting alternate GEE models using unstructured correlations did not change overall findings for our cocaine use outcomes. Finally, the lost to follow-up rate of 14% may also be encouraging for feasibility of future trials. These findings may suggest that it is viable to test interventions to address CUD among people who are actively using cocaine and have an interest in reducing their cocaine use. Given the morbidity, mortality and HIV risk associated with CUD, especially among MSM, as well as the lack of pharmacological interventions for CUD, these findings provide helpful information in addressing this public health issue.

Our pilot study was not powered to detect treatment effects between lorcaserin and placebo on cocaine use and sexual risk outcomes. In addition, because of the early termination of our study, we did not reach our target sample size, further reducing study power. While we did not observe a statistically significant treatment effect in urine positivity for cocaine, we observed a significant protective effect on cocaine use by self-report in timeline follow-back assessments in favor of lorcaserin. Although the protective effect from lorcaserin on self-reported cocaine use observed may be subject to bias, the double-blind study design may have helped mitigate this concern [81]. Furthermore, as noted, we did not observe evidence of unblinding between the two groups (i.e., because participants didn’t know which group they were assigned to, we have no reason to believe that self-report due to social desirability or recall bias would be systematically different between groups). As previously noted, another possible explanation for the different results observed between the two cocaine measures is that the window for cocaine use in the weekly urine tests—estimated to be between 1 to 3 days—was too narrow to detect cocaine use from the prior week [74]. Hence, we may have had non-differential misclassification of cocaine use during weeks when participants used cocaine outside the urine detection window, which would bias the results toward to null [82].

The treatment effect of lorcaserin on the self-reported use of cocaine in our study can be considered in parallel with a human laboratory study finding that lorcaserin compared to placebo significantly reduced craving for cocaine and delayed cocaine administration [52]. However, the negative findings from the recently completed NIDA Clinical Trials Network (CTN) study of lorcaserin for CUD (Clinical Trials.gov NCT03007394) do not lend support to its utility to achieve abstinence (data not yet peer-reviewed, but overall findings described in [83]). That said, the primary outcome of this CTN lorcaserin trial was “Actual Number of Subjects That Successfully Achieve Abstinence From Cocaine During the Last Three Weeks of Treatment”; it did not report whether lorcaserin was associated with reductions in cocaine use [83]. Nevertheless, because lorcaserin is no longer commercially available (the manufacturer has removed it from the market voluntarily at the request of the FDA), our findings may be viewed more generally as potential evidence in support of future medications with similar mechanisms of action as lorcaserin (i.e., other selective serotonin agonists or other 5-HT_2C_R agents [84]). As noted, there are currently no FDA-approved pharmacotherapies for CUD. Hence, as novel pharmacologic agents with similar mechanisms as lorcaserin are identified and deemed safe for use, it may be important for future research to examine their therapeutic potential for CUD. Furthermore, given the linkages between cocaine use and HIV-related sexual risk behaviors and HIV care outcomes, conducting future trials of pharmacotherapies could aid in the development of efficacious strategies for CUD that can also jointly address HIV transmission and acquisition.

This pilot study has several limitations. As mentioned, this study was not originally powered to assess the efficacy of lorcaserin versus placebo, and because the study was terminated early, the study was likely further under-powered in these efficacy analyses. These should be kept in mind while interpreting the null findings in exploratory analyses between treatment groups for cocaine urine-positivity and sexual risk behaviors. Additionally, as noted above the measures on self-reported cocaine use is subject to non-differential misclassification, which is more likely to lead to bias toward the null [82]—it is possible that the effect of lorcaserin may be larger with more precise objective measures. Moreover, quantitative urine analysis could enhance the ability to detect changes in cocaine use that are otherwise missed in qualitative urine screens [85]. Furthermore, given the exploratory nature of the between-group analyses, we did not formally control for multiple hypotheses testing, therefore findings should also be interpreted with caution, particularly those with wide confidence intervals. Additionally, the early termination of our study, in combination with our 2:1 treatment assignment resulted in imbalanced demographic characteristics in the trial, which may hinder external validity. Finally, our small sample size and use of non-probability sampling, and study population of cisgender MSM limits the generalizability of our findings.

Despite these limitations, we found that it is feasible, acceptable, and tolerable to conduct a placebo-controlled pharmacologic trial for MSM with CUD who are actively using, with strong attendance, compliance with study activities, and retention. In this pilot study, lorcaserin, a 5-HT_2c_R agonist, was not associated with significant reductions in cocaine urine positivity, but it was associated with significant reductions in self-reported cocaine use. Future research is needed to continue to explore the potential utility of 5-HT_2c_R agonists. Given the public health importance of CUD, the lack of FDA-approved pharmacologic interventions, and cocaine’s linkages to HIV, efforts to evaluate other cocaine interventions are urgently needed.

## Supporting information

S1 File Consort checklist. This is the CONSORT checklist for this clinical trial.
(DOC)

S2 File Study protocol. This is the protocol for this clinical trial.
(PDF)
