# Supplementary material for: Pilot study of extended-release lorcaserin for cocaine use disorder among men who have sex with men: A double-blind, placebo-controlled randomized trial
Source: PLoS One. 2021 Jul 15;16(7):e0254724. doi: 10.1371/journal.pone.0254724 (PMC8282062; doi:10.1371/journal.pone.0254724)
Supplement: S2 File — This is the protocol for this clinical trial. (PDF) [file pone.0254724.s002.pdf]

Study Title:

TREATMENT WITH LORCASERIN FOR COCAINE USE: THE TLC STUDY

INVESTIGATIONAL NEW DRUG APPLICATION

AND

CLINICAL STUDY PROTOCOL

**Study Director:**	Name:	Glenn-Milo Santos, PhD, MPH
	Telephone:	415-437-6132
	E-mail:	Glenn-Milo.Santos@ucsf.edu

**Protocol Version/Date:**	Original:	08 November 2017

2.0 Table of Contents

2.0 Table of Contents 1

3.0 INTRODUCTORY STATEMENT AND GENERAL INVESTIGATIONAL PLAN 4

3.1 Introductory Statement 4

3.1.1 Drug Name and Active Ingredients 4

3.1.2 Pharmacological Class 4

3.1.3 Structural and Chemical Formula 4

3.1.4 Formulation of Dosage 4

3.1.5 Route of Administration 5

3.1.6 Broad Objectives and Planned Duration of the Proposed Clinical Investigation 5

3.2 Brief Summary of Previous Human Experience 5

3.3 Withdrawn From Investigation or Marketing in any Country 6

4.1 GENERAL INVESTIGATIONAL PLAN 6

4.2 Rationale for Drug and Study 6

4.3 General Approach to be Followed in Evaluating the Drug 8

5.0 Package Insert 9

6.0 PROTOCOL 10

6.1 Objectives and Purpose of Study 10

6.1.1 Specific Aims 10

6.2 Investigator and Facilities Data 10

6.2.1 Form FDA 1572 10

6.2.2 Sponsor-Investigator 11

6.2.3 Sub-investigator(s) 11

6.2.3.1 Form FDA 3454 11

6.3 Patient Selection and Estimated Number to be Studied 11

6.3.1 Inclusion and Exclusion Criteria 11

6.4 Description of Study Design 12

6.4.1 Prescreening 13

6.4.2 Consent 13

6.4.3 Screening 13

6.4.4 Randomization 14

6.5 Drug Dosing- Method, Maximum Dose, and Duration of Exposure 14

6.5.1 Method 14
6.5.2 Maximum Dose 14
6.5.3 Duration of Exposure 14
6.6 Observations and Measurements Made to Fulfill Study Objectives 14
6.6.1 Adherence 15
6.6.2 Ecological Momentary Assessments (EMA) Text Messages 15
6.6.3 Urinalysis for Cocaine Use 15
6.6.4 Sweat Testing 16
6.6.5 Qualitative Interviews 16
6.6.6 Behavioral Survey Measurements 17
6.6.7 Electrocardiogram (ECG) 17
6.7 Procedures to Monitor Drug Effects in Human Subjects and Minimize Risks 17
6.7.1 Potential Medication Risks 17
6.7.2 Serotonin Syndrome Risks 18
6.7.3 Venipuncture Risks 20
6.7.4 Risk to Privacy and Confidentiality 20
6.7.5 Electrocardiogram (ECG) Risks 21
6.7.6 Randomization Risks 21
6.7.7 Adverse Event Reporting 21
6.8 Clinical Trial Registration 22
7.0 CHEMISTRY, MANUFACTURING AND CONTROL INFORMATION 23
7.1 Chemistry 23
7.2 Manufacture 24
7.2.1 Purity 24
7.2.2 Investigational Drug Label 25
7.2.3 Claim for Categorical Exclusion for Environmental Assessment 26
8.0 PHARMACOLOGY AND TOXICOLOGY INFORMATION 27
9.0 PREVIOUS HUMAN EXPERIENCE 28
10.0 ADDITIONAL INFORMATION 29
11.0 RELEVANT INFORMATION 30
12.1 APPENDICES COVER SHEET 31
12.2 Appendix 1: Cover Sheet 32
12.3 Appendix 2a and 2b and 2c: Cover Sheet 44

12.4 Appendix 3a-3c: Cover Sheet 49
12.5 Appendix 4: Cover Sheet 94
12.6 Appendix 5: Cover Sheet 97
12.7 Appendix 6: Cover Sheet 124
12.8 Appendix 7: Cover Sheet 165
12.9 Appendix 8: Cover Sheet 167

3.0 INTRODUCTORY STATEMENT AND GENERAL INVESTIGATIONAL PLAN

Study Title:
TREATMENT WITH LORCASERIN FOR COCAINE USE: THE TLC STUDY

3.1 Introductory Statement

The study conducted under this Investigational New Drug Application will utilize BELVIQ® XR (lorcaserin), manufactured by: Arena Pharmaceuticals: Untere Brühlstrasse 4, 4800 Zofingen, Switzerland

The overall goal of the study is to evaluate the feasibility, tolerability, acceptability and adherence for lorcaserin among actively using men who have sex with men (MSM) with cocaine use disorders. We propose a pilot double-blinded, placebo-controlled trial to study these aims among MSM.

- The proposed clinical investigation is not being conducted to support a label-change of the product or to support a significant change in the marketing of the product.
- The investigation does not involve a change in the dose or route of administration and is FDA-approved.
- This investigation may put the study population at higher risk for serotonin syndrome due to concomitant use of cocaine, leading to the application for an IND.

3.1.1 Drug Name and Active Ingredients

Name(s): BELVIQ® XR

Active Ingredients: lorcaserin hydrochloride

3.1.2 Pharmacological Class

BELVIQ® XR (lorcaserin hydrochloride) is a serotonin 2C receptor agonist for oral administration used for chronic weight management. [1]

3.1.3 Structural and Chemical Formula

Its chemical name is (R)-8-chloro-1-methyl-2,3,4,5-tetrahydro-1H-3-benzazepine hydrochloride hemihydrate. The empirical formula is C H Cl N·0.5H O, and the molecular weight of the hemihydrate form is 241.16 g/mol.[1]

The structural formula is:

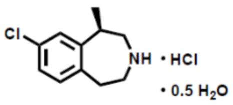

3.1.4 Formulation of Dosage

This drug is in the form of an orange, film-coated, round, film-coated, 20 mg extended release tablet. The tablets are biconvex, debossed with “A” on one side and “20” on the other side.

For this investigation, BELVIQ® XR will not be compounded. We will have Safeway Pharmacy (located at 6100 Hellyer Avenue, Suite 100 San Jose, CA 95138) encapsulate the BELVIQ® XR pill in whole form and create identical placebo capsules.[1]

3.1.5 Route of Administration

The recommended dose of BELVIQ® XR is one 20mg tablet administered orally once daily.[1]

3.1.6 Broad Objectives and Planned Duration of the Proposed Clinical Investigation

The proposed clinical investigation is a placebo-controlled, double-blind 12 week trial evaluating the feasibility, tolerability, acceptability and adherence for lorcaserin among actively using, men who have sex with men (MSM) with cocaine use disorders. This investigation is proposed to take 2.5 years and will enroll 45 individuals who will be randomly assigned to either the treatment (lorcaserin) arm (n=30) or the placebo arm (n=15), to be taken once daily.

3.2 Brief Summary of Previous Human Experience

Data from other preclinical and clinical studies also support lorcaserin's potential to treat drug disorders. For example, lorcaserin has been shown to reduce nicotine self-administration,[2] as well as nicotine discrimination, and cue-induced reinstatement of nicotine seeking.[3] *Currently, another team is evaluating lorcaserin in a placebo-controlled Phase 2, 12-week trial on smoking cessation* (ClinicalTrials.gov #: NCT02044874). Additionally, there is an ongoing human laboratory study evaluating the effect of lorcaserin in craving and intravenous cocaine self-administration within a laboratory setting (NCT02680288). Moreover, lorcaserin's proven clinical efficacy on obesity—and the known neurobiological commonalities between obesity and drug addiction—further raise the potential of lorcaserin in treating cocaine use.[4, 5] Indeed, as highlighted by the director of the National Institute on Drug Abuse (NIDA), Dr. Nora Volkow, obesity and drug addiction share many common molecular, cellular, and systems-level mechanisms, suggesting the possibility that effective treatments for excessive food consumption may have similar effects for excessive drug consumption.[5-8] Lorcaserin may also lead to reductions in cocaine use by reducing impulsivity. Excess impulsivity has long been associated with drug abuse and cocaine use induces greater impulsivity.[9] 5-HT_{2C}R agonists have reduced impulsive action[9, 10] and lorcaserin has specifically decreased nicotine-induced impulsive action in animal studies.[3] We hypothesize that lorcaserin's *dual effects of inhibiting dopamine levels and decreasing impulsivity* may effectively help MSM reduce or stop their cocaine use.

Lorcaserin is an FDA-approved medication that is well-tolerated with relatively few adverse events (AEs).[11] Three Phase 3 trials (cumulative N=7789) that evaluated lorcaserin's treatment effect on obesity have established its safety among patients with and without diabetes.[1, 12-14] The most common reported side effects for lorcaserin include headaches, dizziness, fatigue, nausea, dry mouth, and constipation.[1, 12-14] In these trials, 8.6% of individuals given lorcaserin discontinued treatment as a result of AEs, compared to 6.7% individuals given placebo.[1, 12-14] Aside from pregnancy, there are no contraindications for lorcaserin.[1] Nevertheless, the safety and tolerability of lorcaserin among active cocaine users is unknown. Lorcaserin has a low potential for abuse.[15] The frequency of AEs associated with lorcaserin increases when taken above the therapeutic dose, which may limit abuse potential.[1, 15] A double-blind randomized crossover study to test abuse liability found that lorcaserin was

similar to placebo with respect to subjective liking, overall liking, and ratings on desire to take the drug again.[15] Exceeding the recommended lorcaserin dose levels (20 mg, once daily) were associated with higher levels of known unpleasant side effects and subjective dislike, compared to placebo.[15] Thus, lorcaserin dose escalation would result in primarily negative subjective effects; these self-regulating properties will likely limit the medication's abuse liability.[7, 15]

3.3 Withdrawn From Investigation or Marketing in any Country

We are not aware of any incidences where BELVIQ® XR has been withdrawn from an investigation or marketing in any country.

4.0 GENERAL INVESTIGATIONAL PLAN

4.1 Rationale for Drug and Study

Cocaine use in the US is an *unrelenting public health problem* with serious negative medical, societal, and economic impacts.[16-18] The US is the world's largest consumer of cocaine, accounting for nearly 36% of the total global consumption.[19, 20] In both powder and rock (i.e., "crack") form, cocaine is an addictive psychostimulant; it is estimated that up to one in six cocaine users may develop dependence/moderate-to-severe use disorders.[17, 21, 22] Cocaine is the leading cause of illicit substance–related emergency department visits in the US[18] and it is associated with direct cardiac toxicity, myocardial infarctions and, sudden cardiac death.[23-27] Cocaine use is a major public health issue among men who have sex with men (MSM). National HIV Behavioral Surveillance (NHBS) data indicate that 37% of MSM in the US used cocaine in the past 12 months, which is 15 times more prevalent than the general population.[28, 29] Cocaine use has been increasing among MSM. In San Francisco, cocaine use (past 12 months) climbed from 19% in 2003–2005 to 35% in 2014 (see Figure 1).[30, 31] This increase corresponded with a significant decrease (test for trend p<0.001)[31, 32] in methamphetamine use among San Francisco MSM (from 22% to 13%). San Francisco community-based surveys observed similar trends: cocaine use among MSM increased from 9.6% in 2009 to 20.4% in 2013.[33, 34] Of the estimated 19,657 illicit psychostimulant-using MSM in San Francisco, 81.3% are cocaine users.[30]

Fig. 1 - Stimulant use among MSM, NHBS San Francisco 2003–14

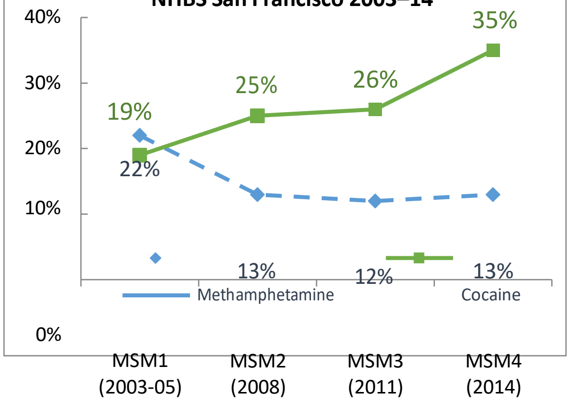

MSM remain disproportionately impacted by HIV—despite sizable efforts to stem the epidemic in this group—accounting for 61% and 90% of new infections in the US and San Francisco, respectively.[35-38] It is imperative to develop more evidence-based HIV interventions for MSM; the National HIV/AIDS Strategy specifically calls for additional interventions for this population.[39] Cocaine use is associated with HIV-related sexual risk behaviors in MSM. In NHBS, 54% of cocaine users reported having sex while under the influence of cocaine.[28] The acute pharmacological effects of cocaine (e.g., altered cognition, impairment of judgment, and

increased sexual desire and confidence) have been attributed to the association between cocaine use and sexual risk behaviors.[40-44] Moreover, a myriad of psychosocial factors (e.g., cognitive escape, impulsivity, and expectancies) are thought to account for the link between cocaine use and sexual risks.[45-48] Furthermore, the bar, club, and circuit-party (weekend-long dance events) settings frequented by MSM are environments conducive to both cocaine use and meeting multiple sex partners.[49-51] Indeed, cocaine use is independently associated with unprotected anal intercourse, multiple partners, increased duration of sex, anonymous partners, sex in a public sex venue, and exchanging money or drugs for sex.[52-57] In the Project ECHO study, a large San Francisco study of substance-using MSM conducted by the proposed research team, *increased frequency of cocaine use* was associated with greater HIV-related sexual risk; those who reported episodic use (less than weekly) and those with weekly or greater cocaine use had 1.86- and 3.13-fold greater odds of HIV serodiscordant unprotected anal intercourse, respectively, compared to non-users.[58] This dose-dependent relationship is consistent with findings from a probability sample of HIV-negative MSM; those with cocaine dependence were more likely to have high-risk sexual behaviors than their non-dependent counterparts.[55]

Cocaine use has been significantly associated with new HIV infections.[59-61] In the EXPLORE longitudinal study of 4,295 HIV-negative MSM from six cities, cocaine use was associated with a 2.24-fold greater hazard for HIV seroconversion.[60] Similarly, in the Multicenter AIDS Cohort Study, use of cocaine or other stimulants, alone or in combination with other drugs, was attributed to 37.3% of the new HIV infections in the study.[59] In a probability sample of San Francisco MSM, cocaine and other stimulant use was also 2.5-fold more common among MSM living with HIV.[62] Among HIV-positive individuals, cocaine use was associated with poor adherence to antiretroviral treatment (ART), HIV disease progression, lower CD4 count, and higher mortality.[63-66] Weekly cocaine use was associated with higher HIV viral load, even after controlling for ART adherence, [67] and in *in vitro* studies, cocaine enhanced HIV viral replication.[68, 69] Poor ART adherence and higher HIV viral load among cocaine users may potentiate greater risk of HIV transmission.[70]

Innovative treatments for cocaine use disorders are needed for MSM.[71] Because cocaine use is common among MSM and is associated with high-risk sexual behavior and HIV infection, interventions that can reduce cocaine use are likely to have an impact on reducing the transmission of HIV.[42] Current prevention approaches to cocaine use for MSM are therapy and substance use treatment programs. However, most substance-using MSM do not access drug treatment, reinforcing the need to develop interventions outside of treatment settings.[58, 72, 73] Furthermore, while some behavioral interventions for substance-using MSM report reductions in both substance use and HIV risk behaviors,[74, 75] behavioral interventions alone have limited efficacy and may benefit from adjuvant pharmacologic agents.[71, 76] However, there are no FDA-approved medications for cocaine use disorders, which severely limits treatment options.[77, 78] Pharmacologic approaches have been successful in treating nicotine, alcohol, and heroin dependence.[79-81] Despite the link between cocaine use and HIV risk, the use of pharmacologic interventions to reduce cocaine-related sexual risk among MSM remains underexplored. The absence of published studies on efficacious interventions to reduce cocaine-related sexual risk behaviors among MSM who have no desire to abstain from cocaine *presents a major gap in the field of substance use research and HIV prevention.* Pharmacotherapy for methamphetamine dependence in MSM has shown that reductions in substance use can result

in parallel reductions in methamphetamine-related sexual risk behaviors among MSM not seeking treatment.[82] Pharmacotherapy for cocaine use may lead to an analogous parallel reduction in cocaine-related sexual risks.

Lorcaserin, a novel selective serotonin (5-HT) receptor agonist, is a promising agent to treat cocaine use disorders. Cocaine use increases dopamine (DA) levels, the neurochemical responsible for the reinforcing effects of the drug, and there is compelling data suggesting that the DA system's activity is modulated by the central 5-HT systems.[7, 83, 84] In particular, the 5-HT_{2C} receptor (5-HT_{2C}R) has been identified as the primary 5-HT receptor subtype that inhibits the DA system's activity.[84] 5-HT_{2C}R agonists have reduced dopamine release in the nucleus accumbens (NAcc) and frontal neurocortex.[83, 84] Conversely, increased DA release in the NAcc result from the blockade of 5-HT_{2C}R.[85] Furthermore, genetic variations in the 5-HT_{2C}R and its functional status have been associated with vulnerability to cocaine cue reactivity—a strong predictor of relapse—and relapse-like behaviors in cocaine dependent rodents and humans.[86, 87] *These data lend support to the use of agents that selectively activate the 5-HT_{2C}R.* We postulate that lorcaserin's mechanism of action as a potent 5-HT_{2C}R selective agonist will have inhibitory control on DA activity, which may decrease the positive rewarding effects of cocaine and lead to reductions in use, while 5-HT_{2C}R activation itself may lead to reductions in likelihood of relapse. Preclinical data support the potential of lorcaserin in reducing cocaine use.[7, 88-98] Lorcaserin is 18- and 120-fold more selective toward the 5-HT_{2C}R compared to 5-HT_{2A} and 5-HT_{2B} receptors, respectively.[99] 5-HT_{2C}R selective agonists, including lorcaserin, have been associated with reductions in cocaine self-administration in mice[100, 101] and non-human primates.[97, 102] Consistent reductions in cue- and cocaine-primed drug seeking have resulted from use of 5-HT_{2C}R selective agonists.[92, 100, 103, 104] Furthermore, 5-HT_{2C}R selective agonists have attenuated acute cocaine-induced activity and the development of long-term cocaine-induced locomotor sensitization.[91] Reinstatement studies to model the relapse processes have also found that 5-HT_{2C}R selective agonists reduced cocaine seeking behavior, again in both rodents [92, 100, 104-107] and primates.[97]

4.2 General Approach to be Followed in Evaluating the Drug

To evaluate the use of lorcaserin among participants with cocaine use disorder, the following methods will be used:

- To explore the *tolerability* of lorcaserin versus placebo among MSM with cocaine use disorders, as determined by the number of adverse clinical events in the cocaine and placebo arms. We will compute the proportions of those experiencing adverse events, both overall and by type. Adverse clinical events, AEs and SAEs, and other binary safety outcomes will be presented as percent of participants that experience the safety outcome by treatment assignment. We will also classify AEs as any AE that results in stopping study medication, specific common AEs (>2% in both groups), AEs for general organ system categories, and AEs potentially related to study drug. The probability of observing at least one AE with population incidence of 1, 2, and 5% in the active arm will be 26, 45, and 70% with 2:1 randomization, respectively.
- To evaluate the *preliminary efficacy* of lorcaserin versus placebo to reduce cocaine use among MSM with cocaine use disorders, as determined by the proportion of cocaine-

positivity from weekly urine samples. Minimum detectable effects (MDE) of the intervention on cocaine use outcomes will be substantial, as is typical of a pilot study. In our prior trial for MSM actively using stimulants, [82] the within-subject correlation of the repeated measures for urine positivity was 0.45, the urine positivity in the control group was 55%, and the retention was 93% at the end of the study. On this basis, we conservatively estimate that the sample will provide 80% power to detect reductions of 29 percentage points in the rate of cocaine urine positivity. In preparation for a larger trial, we will conduct exploratory assessments using GEE Poisson models to assess treatment effects on cocaine urine positivity.[108]

- **Exploratory Aims:** *Mechanism of lorcaserin to reduce cocaine use:* We will use linear mixed models to explore whether lorcaserin reduces impulsivity as measured by BART[109] and the STIMP[110], and sexual impulsivity.[111] We will also assess whether lorcaserin reduces cocaine craving[112, 113] and severity of cocaine dependence,[114] using similar methods. *Exploratory mediation analyses:* Our ability to detect evidence of mediation will be limited in this pilot study. However, in preparation for a larger trial, we will conduct exploratory assessments; isolated findings from these exploratory assessments will be interpreted with caution. We will explore whether changes in the mediators listed above (e.g., impulsivity, craving, severity of dependence) are associated with current or later reductions in cocaine use and cocaine-related sexual behaviors, using GEE models with robust standard errors controlling for treatment as well as confounders of the mediator-outcome relationship.[115] *Evidence of unblinding:* We will tabulate the proportions of participants in each arm who correctly guess their treatment assignment, and determine if there is significant evidence of unblinding at the end of the trial using Fisher's exact tests. *Consecutive weeks abstinent*: There is increasing interest in looking at consecutive weeks of continued cocaine abstinence (termed, by NIDA, as "number of beyond threshold weeks of success" (NOBWOS))[116] as part of pharmaceutical interventions, and NIDA now requires this outcome in their pharmaceutical trials. We will use Wilcoxon rank-sum tests to compare NOBWOS in the 0–2 month intervals. We will not use this as our primary outcome because risk-reduction approaches (which include, but are not limited to abstinence) are of high public health benefit, affect a broader segment of the cocaine-using population, many of whom are not prepared to commit to abstinence, and because our original trial showed benefit using a risk-reduction approach.

5.0 Package Insert

Please see the BELVIQ® XR Package Insert in Appendix 1.

6.0 PROTOCOL

6.1 Objectives and Purpose of Study

The proposed clinical investigation is a placebo-controlled, double-blind 12 week trial evaluating the feasibility, tolerability, acceptability and adherence for lorcaserin among actively using, men who have sex with men (MSM) with cocaine use disorders. This study will represent the first Phase 2 randomized outpatient clinical trial to evaluate the potential of lorcaserin to reduce cocaine use among actively using participants with cocaine use disorders. Although there is an ongoing human laboratory study of lorcaserin's effects on craving and intravenous cocaine self-administration during overnight confinements at a medical center (ClinicalTrials.gov #: NCT02680288), our proposed study will provide distinct yet complementary data on the viability of lorcaserin to reduce cocaine. Our study is unique because it involves the outpatient treatment and evaluation of lorcaserin to reduce cocaine use and cocaine-associated sexual risk behaviors over a 12-week follow-up period. Hence, data from this pilot can inform the sample size required for a larger clinical efficacy trial of lorcaserin for outpatient treatment of cocaine use.

6.1.1 Specific Aims

1. To establish the feasibility of enrolling and retaining 45 MSM with cocaine use disorders in a 12-week randomized, double-blind study of lorcaserin versus placebo.

2. To assess the tolerability of lorcaserin versus placebo among MSM with cocaine use disorders, as determined by the number of adverse clinical events in the lorcaserin and placebo arms.

3. To establish the acceptability and adherence for lorcaserin versus placebo among MSM with cocaine use disorders, as determined by qualitative interviews and evaluations on study procedures, study drug characteristics and dosing schedule and by medication adherence to lorcaserin and placebo.

4. To evaluate the preliminary efficacy of lorcaserin versus placebo to reduce cocaine use and HIV-related sexual risk behaviors among MSM with cocaine use disorders, as determined by the proportion of cocaine-positivity from once weekly urine samples and self-reported sexual behaviors, by treatment arm.

6.2 Investigator and Facilities Data

6.2.1 Form FDA 1572

Please see the signed and dated FDA Form 1572 in Appendix 4.

6.2.2 Sponsor-Investigator
Please see the Investigator’s CV in Appendix 5.

6.2.3 Sub-investigator(s)
The CVs of the sub-investigator listed below may be found in Appendix 6.

Phillip O. Coffin, MD

6.2.3.1 Form FDA 3454
Please see the completed Form FDA 3454 in Appendix 7.

6.3 Patient Selection and Estimated Number to be Studied
We will recruit 45 participants who will be seen at our office on 25 Van Ness Avenue, San Francisco, CA. Recruitment will be through advertisements, active recruitment and snowball sampling. Recruited individuals will be asked a brief general eligibility questionnaire in person out in the field or over the phone by research staff after providing verbal consent. Once a prospective subject has been identified, research staff will conduct a telephone interview to assess possible eligibility. Participants who have phone-screened eligible will be scheduled for an in-person visit where consenting and further eligibility will be assessed via clinical examination, laboratory safety testing, urine drug test for cocaine metabolites and cocaine frequency, prior to being randomized.

6.3.1 Inclusion and Exclusion Criteria
Inclusion Criteria:

1) Male gender assigned at birth and transgender men;
2) Age 18 - 65 years
3) Have the ability to understand, and having understood, provide written informed consent to comply with the treatment protocol
4) self-reported anal intercourse with men in the prior six months
5) meets Diagnostic and Statistical Manual of Mental Disorders, Fifth Edition, Text Revision (DSM- V-TR) diagnostic criteria for cocaine use disorder (CoCUD) as determined by the Structured Clinical Interview (SCID);
6) has used cocaine on at least 1 day in the last 30 days prior to screening, confirmed by urinalysis at screening (at least one sample positive for cocaine metabolites within 2 screening and 2 run-in visits, from an on-site, qualitative benzoylecgonine (BE) assay (urine dipstick));
7) HIV-negative by rapid test <u>or</u> HIV-positive with medical record of HIV infection and with a CD4 count greater than 200 cells/mm3;
8) no current acute illnesses requiring prolonged medical care;
9) no chronic illnesses (somatic and mental) that are likely to progress clinically during trial; Chronic stable illnesses will be reviewed on a case by case basis by the study clinician for eligibility;
10) baseline CBC, total protein, albumin, glucose, alkaline phosphatase, creatinine, BUN, and electrolytes without clinically significant abnormalities as determined by study clinician in conjunction with symptoms, physical exam, and medical history. (Please see "Medical Safety Lab Assessments_022717" under the "Other Study documents" section for a reference gide to lab test

Exclusion Criteria:

1) Any current psychiatric condition (e.g., depression with suicidal ideation, schizophrenia) or medical condition that would preclude safe study participation; or any history of head trauma with sequelae, dementia, or organic brain disease;
2) HIV-positive test result at screening visit but previously unaware of HIV infection (i.e., newly diagnosed with HIV infection at screening; those with a medical record of HIV infection are eligible);
3) any current severe drug use disorders (other than cocaine, nicotine or cannabis use disorders), according to DSM-V criteria;
4) known allergy or previous adverse reaction to lorcaserin;
5) current CD4 count < 200 cells/mm^3 ;
6) moderate/sever liver disease (AST, ALT > 3 times upper limit or normal);
7) severely impaired renal function (creatinine clearance £ 30 ml/min);
8) use of medications that affect the serotonergic neurotransmitter system (e.g., selective serotonin reuptake inhibitors (SSRIs), selective serotonin-norepinephrine reuptake inhibitors (SNRIs), tricyclic antidepressants, monoamine oxidase inhibitors (MAOIs));
9) predisposition to priapism;
10) currently participating in another longitudinal intervention research study;
11) body mass index (BMI) < 15; or ≥ 30 with desire to use weight management medication, *or* BMI > 35;
12) anticipated use of agents that are associated with valvulopathy and/or pulmonary hypertension
13) Are currently treated with an opiate-substitute (buprenorphine or methadone) maintenance treatment or received therapy with any opiate-substitute within 30 days preceding screening
14) Currently in court-mandated drug treatment;
15) Had previous history of suicidal behavior in the last 12 months ("yes" answer to the suicidal behavior question 6 of the Columbia-Suicide Severity Rating Scale (C-SSRS)); or currently have suicidal ideation as determined by 'yes' answers to questions 4 or 5 on the C-SSRS administered by a study clinician;
16) Any physical condition affecting drug absorption (e.g., gastrectomy);
17) 12-lead ECG demonstrating QTc > 450 or a QRS interval > 120 msec at screening. If QTcF exceeds 450 msec, or QRS exceeds 120 msec, the ECG should be repeated two more times and the average of the three QTc values should be used to determine the subject's eligibility.
18) Current alcohol use disorder that is judged to require supervised medical detoxification;
19) Not seeking treatment for cocaine use

(**Note:** Participants newly diagnosed with HIV at screening who consent to be contacted for re-screening will be called in the subsequent month, or later, depending on participant preference).

6.4 Description of Study Design

This placebo-controlled, double-blind clinical trial has two phases: a 2-visit screening assessment and 12-weeks of treatment. The 12-week treatment phase will consist of treatment with lorcaserin or placebo and a weekly study visit with research staff at the San Francisco Department of Public Health.

6.4.1 Prescreening

Prospective participants will undergo a telephone pre-screener questionnaire to establish that they meet certain eligibility criteria. Some criteria study staff will be asking about is their age, how often they use cocaine, whether they wish to reduce or stop their cocaine use, and if they have had anal course with a man while under the influence of cocaine in the last 6 months.

6.4.2 Consent

If a potential participant is determined eligible after taking the pre-screener questionnaire, study staff will schedule them to come in for an in-person screening assessment. Before any screening procedures are completed, participants will provide written consent in compliance with IRB regulations. The consent process will discuss the 2:1 random assignment of participants to receive lorcaserin or placebo and will detail the potential adverse effects of lorcaserin. It will also discuss the weekly substance use counseling sessions. The consent process also addresses participant rights, including the voluntary nature of participation and ability to decline without penalty. Mechanisms for maintaining confidentiality will also be discussed, as well as exceptions to confidentiality which is required by law. Participants will be given a copy of the Human Subject's Bill of Rights, along with a copy of the consent form. Participants are given the contact numbers of both the PI and the UCSF IRB to answer questions about the study or one's rights as a human subject. All participants will meet with a study clinician for an additional opportunity to ask questions. Similar to our prior pharmacologic studies, all participants must also correctly complete an "Assessment of Understanding" quiz. The quiz will include 10 true/false study-related questions that assess participants' understanding of basic study concepts, including the unknown efficacy of lorcaserin to reduce cocaine use among MSM, its side effect profile, the randomization process, and the nature of placebos. After the assessment of understanding has been completed and staff are satisfied that the participant is able to give full informed consent (including, but not limited to, a participant completing the quiz with an 80% score or greater and a demonstrated ability to fully understand corrected answers), the consent form will be signed by the participant. The staff member obtaining consent will also sign both the original and the copy as a witness, completing the informed consent process. A copy of the signed form will be given to the client; the original signed consent will be kept in a separate, locked file.

6.4.3 Screening

After consent, subjects will be scheduled for screening assessments. The screening phase is accomplished over two-four screening assessments, with one screening assessment per week. Screening assessments will include: a clinical examination, safety laboratory testing, a urine drug test for cocaine metabolites, a medical history and Structured Clinical Interview DSM-5 (SCID), pregnancy test, if needed, HIV testing and risk reduction counseling, and an electrocardiogram (ECG).

6.4.4 Randomization

The computer-generated randomization code for the study will have variable blocks and a 2:1 allocation ratio for lorcaserin and placebo. Assignment of two-thirds of participants to the lorcaserin arm was specifically chosen to improve our ability to detect adverse events in the active treatment arm, while preserving our ability to assess recruitment in a study in which randomization to placebo is likely. No study staff will have access to the code. In the event that it becomes absolutely necessary to unblind research staff to what study arm a participant was randomized to, the study biostatistician or Safeway Pharmacy will be able to provide this information.

6.5 Drug Dosing- Method, Maximum Dose, and Duration of Exposure

6.5.1 Method

The Safeway Compounding Pharmacy (San Jose, CA), which is currently supplying the study drug for our ongoing trials, will prepare and supply lorcaserin and placebo in identical-looking gel capsules to maintain blinding. The policies and procedures of Safeway Compounding Pharmacy concerning good compounding practices and environmental, instrumental and procedural quality assurance have been approved by the California State Board of Pharmacy. Furthermore, this pharmacy is regulated by the US Food and Drug Administration (FDA). Medications will be distributed in MEMs Cap Dispensers by the study clinician with dosing instructions, randomization number, dispensing date, prescribing clinician, 24-hour phone number, and advisements against drug combinations. The date, time and amount dispensed to the participant each visit will be recorded. Furthermore, any treatment that is returned, lost, or accidentally disposed of will be documented.

6.5.2 Maximum Dose

Participants will be given a week’s supply of 20mg tablets at enrollment and weekly visits; they will be instructed to only one tablet every 24 hours.

6.5.3 Duration of Exposure

After random assignment, participants will receive 12 weeks of treatment with either lorcaserin or placebo.

6.6 Observations and Measurements Made to Fulfill Study Objectives

6.6.1 Adherence

We will collect self-reported adherence *via* modified timeline follow-back (TLFB) [117, 118] assessments and pill counts at weekly visits, and *via* daily text messages. MEMs Cap Dispensers will track adherence daily; each dispenser opening is recorded as a medication event sent to a remote database in real time. MEMs Cap Dispensers have been shown to be reliable for adherence monitoring, and we have successfully used electronic medical monitoring devices in prior studies.

**Table 2. The TLC Study Procedures**	**Scr 1&2**	**Enr**	**Wk**	**Dly**	**M 1&2**	**M 3**
Informed consent	X					
Safety lab assessment	X				X	X
Rapid HIV test*, CD4^, viral load^, pooled VL`	X					X
HIV risk reduction counseling	X					x
Medical history and Structured Clinical Interview DSM-5 (SCID)	X					
Pregnancy test (if needed)	x					
Vital signs, weight	X	X			X	X
Physical exam	x	X	x			X
ECG	X					X
PHQ-2		x	x			
Medication Training		X				
Randomization		X				
Urine testing for cocaine	X	X	X		X	X
Sweat patch testing for cocaine		X	X		X	X
CAPI assessments		X	X		X	X
AE assessments			X		X	X
Substance use counseling			X			
Qualitative Interviews						X
Text Messaging				X		

Notes: *Confirmatory HIV testing will be performed for reactive tests. ^CD4 and VL will be done for HIV-positive individuals only. `Pooled VL will be done for HIV-negative individuals only.
Scr=Screening, Enr=Enrollment, Wk=Weekly, Dly=Daily, M=Monthly

6.6.2 Ecological Momentary Assessments (EMA) Text Messages

Participants will receive daily EMA texts to collect data on cocaine use patterns and medication compliance.[119] Messages will use shorthand notations to maintain participant confidentiality. Participants will be trained on text messaging procedures and receive a reference guide during enrollment. Participants will be encouraged to regularly delete text messages and encrypt their phone with passwords for privacy.

6.6.3 Urinalysis for Cocaine Use

Urine samples will be collected weekly and tested for cocaine metabolites (benzoylecgonine) with EZ-SCREEN screening assays (MedTox Diagnostics, Inc., Burlington, NC). On average, cocaine is detectable in urine for approximately 72 hours. To detect use over weekends, when cocaine use is likely to be higher compared to the remainder of the week, we will schedule at least one of the two urine collection visits on Mondays and Tuesdays each week whenever possible. Within a given week, urine collection visits will be schedule 3-4 days apart (e.g., Mon & Thurs; Mon & Fri; Tue & Fri; Thurs & Mon). Furthermore, the MedTox assays we will use for urine toxicology tests can also detect the following classes of substances: amphetamine, barbiturates, benzodiazepines, buprenorphine, methadone, methamphetamine, opiates, oxycodone, phencyclidine, propoxyphene and THC (cannabinoids).

6.6.4 Sweat Testing

To further enhance our ability to detect cocaine use, we will also utilize tamper evident sweat patches—PharmChek Drugs of Abuse Sweat Patch (PharmChem, Inc., Fort Worth, Texas)—that absorb perspiration 24 hours per day, for up to 10 days of continuous wear.[120] These patches will be tested for the presence of cocaine and cocaine metabolites, using testing procedures approved by the FDA since 1995.[120] Pharmchek patch enzyme immunoassays to detect cocaine in sweat have been found to have 94-95% sensitivity and 91-99% specificity rates, compared to gas chromatography-mass spectrometry.[121, 122] At baseline visit, we will affix patches on all participants. These patches have a semi-permeable membrane to allow oxygen, carbon dioxide and water vapor to pass through the patch, keeping the skin underneath sterile and healthy. At each weekly follow-up visit, we will replace the used patch worn since the prior visit and send those used patches for cocaine testing to PharmChem's laboratories. These patches will retain cocaine and cocaine metabolites for any use during the period it was worn. Hence, we anticipate no gaps in the window of detection for cocaine use. Research staff will follow PharmChem's guidelines for any AEs that may occur while a participant has a patch affixed to their skin.

Extra replacement patches will be provided to participants at weekly visits, in anticipation of the rare event that a patch falls off prematurely; participants will be instructed to use a replacement patch when needed. Used patches will be collected and tested weekly. In sensitivity analyses, we will impute cocaine-positive results for weeks in which multiple patches were used or when patches fell off.

6.6.5 Qualitative Interviews

All participants will be scheduled for in-depth semi-structured interviews at their 12-week treatment visit. Qualitative experts will conduct interviews to explore participant treatment and study acceptability and assess medication adherence challenges. Semi-structured interview guides will begin with a discussion of motivations for treatment seeking and ask about the participant's history of drug use and treatment programs. The guide will then assess overall experiences as part of the study (e.g., frequency of study visits, frequency of medication dosing, appraisal of study activities and staff, study location). Participants who have experience with pharmacologic interventions for substance use treatment will be asked to compare their experiences. The guide will then segue to discuss anticipated challenges to medication adherence (e.g., perceived effects or no effects of the study drug on cocaine cravings, housing instability, depression, alcohol or other drug use, stigma), and ask open-ended questions to explore the context in which participants are participating in this study and to identify other potential barriers (e.g., partner drug and alcohol use). At the end of the interview, the interviewers will review the treatment adherence challenges discussed and ask participants to rank order challenges by most-to-least important.

Interviews will take about 30 minutes. All interviews will be conducted one-on-one and audio-recorded in a private interview space. Qualified study staff will review audio files together in real time for quality assurance and technique. All recorded interviews will be downloaded electronically to a secure network. Only research staff involved with this study will have access

to those electronic files. Once the audio interviews are downloaded, they will be disposed of on the recording device. At the end of the study, the interviews will be transcribed and then destroyed.

6.6.6 Behavioral Survey Measurements

Validated psychometric scales and behavioral measures will be assessed via CAPI to standardize data collection and minimize underreporting of risk behaviors.[123, 124] To better understand the mechanism in which lorcaserin may reduce cocaine and cocaine-associated sexual risk behaviors, we will evaluate lorcaserin's effect on impulsivity through two validated measures of impulsivity: the state impulsivity scale (the STIMP)[110] and the Balloon Analogue Risk Task (BART).[109] We will evaluate changes in cocaine craving by arm using the visual analog scale for craving[112] and the Minnesota cocaine craving scale.[113]

6.6.7 Electrocardiogram (ECG)

A 12-lead ECG will be done to assess participant eligibility during screening and again at study exit to assess safety at termination. The ECG tracings will be reviewed by a qualified medical clinician for accuracy at the site.

6.7 Procedures to Monitor Drug Effects in Human Subjects and Minimize Risks

6.7.1 Potential Medication Risks

A primary risk of participation in this study is the potential of AEs due to taking lorcaserin, an FDA-approved medication for weight management. These risks are seen as reasonable, given the favorable side effect profile for this medication and the fact that we will exclude persons from the trial who are at higher risk for AEs. As part of the informed consent process, participants will be informed of the potential risks of taking lorcaserin.

Lorcaserin is an FDA-approved medication that is well-tolerated and has relatively few adverse events.[11] Three placebo-controlled phase III trials (cumulative sample size = 7789) have established its safety among patients with and without diabetes.[1, 12-14] The most common side-effects for lorcaserin reported include headaches, dizziness, fatigue, nausea, dry mouth, and constipation.[1, 12-14] In these trials, 8.6% of individuals given lorcaserin discontinued treatment as a result of AEs, compared to 6.7% individuals given placebo.[1, 12-14]

Aside from pregnancy, there are no contraindications for lorcaserin.[1] Among patients who received lorcaserin, 1.9% reported impairments in attention and memory, compared to 0.5% of those receiving placebo.[11] Participants will be cautioned against operating hazardous machinery until they know that they will not experience cognitive impairment. In clinical trials, 1.3% of individuals receiving lorcaserin discontinued the medication due to depression, mood disorders, or suicide ideation, compared to 0.6% of individuals on placebo. As with our other pharmacologic studies, clinicians will administer the patient health questionnaire-2 (PHQ-2) at weekly visits to inquire about frequency of depressed mood and anhedonia. Participants with

PHQ-2 score of 3 or more will be screened further for depression. Participants who develop symptomatic depression (or have worsening depressive symptoms compared to baseline) will be taken off study medication. Patients who experience suicidal thoughts will be taken off study medication. As indicated, participants with type 2 diabetes will have their blood glucose levels monitored for hypoglycemia during the course of the trial; diabetic participants will be taken of study medication if needed.[11] Levels of prolactin will be measured among patients who report symptoms of hyperprolactinemia; those with prolactin levels above the upper limit of normal will be taken off study medication if needed.

Lorcaserin has a low potential for abuse.[15] The frequency of side effects associated with lorcaserin increases when taken above the therapeutic dose, which may limit abuse potential.[1, 15] A double-blind, placebo controlled, double-dummy, randomized crossover study to test abuse liability found that lorcaserin was similar to placebo with respect to levels of subjective liking, overall liking, and ratings desire to take the drug again (unlike other agents tested—zolpidem and Ketamine—which both significantly exceeded subjective effects recorded for placebo).[15] In addition, lorcaserin intake exceeding the recommended levels for treatment was associated with significantly higher levels of known unpleasant side effects and subjective dislike for the lorcaserin compared to zolpidem, ketamine, and placebo.[15] Hence, these findings suggest that escalation of lorcaserin doses will be associated with primarily negative subjective effects, limiting potential for abuse.[8, 15] Lorcaserin has an 18-fold greater affinity to the $5HT_{2c}$ receptor at therapeutic levels (two 10 mg tablets daily or 20 mgs per day). Doses exceeding 20 mg per day are associated with increased side effects.[15] Doses above 20 mg may also be associated with euphoria and hallucinations in short-term studies. Participants will be advised not to exceed the therapeutic dose of 20 mg (20 mg once daily).

Lorcaserin is also contraindicated in patients who are pregnant. Given the study's target population (self-identified men who have sex with men), the team anticipates that few participants will be excluded by virtue of pregnancy. Transgender participants will be screened for pregnancy prior to enrollment. The package insert for lorcaserin contains cautions against severe liver impairment; severely impaired renal function; use of medications that affect the serotonergic neurotransmitter system (e.g., selective serotonin reuptake inhibitors (SSRIs), selective serotonin-norepinephrine reuptake inhibitors (SNRIs); tricyclic antidepressants; monoamine oxidase inhibitors (MAOIs)); predisposition to priapisms; and use of any agents that are associated with valvulopathy and/or pulmonary hypertension. For the proposed study, we will exclude any individuals with these cautions.

6.7.2 Serotonin Syndrome Risks

There have been no cases of serotonin syndrome documented in clinical trials of lorcaserin.[125] However, <1% of participants have reported symptoms of serotonergic excess (e.g., tremors, chills, confusional state, disorientation, and hyperhidrosis).[125] In the proposed study, we will perform physical examinations focused on assessing mental status change, and autonomic and neuromuscular hyperactivity to screen participants for emergent signs and symptoms of serotonin syndrome at weekly visits.[126] Additionally, study clinicians will

document, on an ongoing basis, all current and recent medications use, including licit and illicit substances, to identify known drugs and drug combinations that cause serotonin syndrome. Participants with signs or symptoms of serotonin syndrome and/or who report use of at least one serotonergic agent within the past 5 weeks will undergo further evaluation for clinically significant serotonin toxicity or serotonin syndrome using the Hunter serotonin toxicity criteria.[126, 127] Those diagnosed with serotonin syndrome will be taken off study medication immediately.

A. AE Reporting

Each AE will be classified by the study clinician as serious or non-serious, and appropriate reporting procedures will be followed; these decisions will be reviewed on a real-time basis by the study clinicians. An AE is defined as any reaction, side effect, or untoward event that occurs during the course of the clinical trial, whether or not the event is considered medication-related. A new illness, symptom, or worsening of a pre-existing condition or abnormality is considered an AE. Stable chronic conditions that are present prior to clinical trial entry and do not worsen are not considered AEs. For this study, AEs will include symptoms reported by the patient and abnormal measures of clinical importance noted by study staff.

Study staff will assess participants for any medical or psychiatric side effects by asking the participant, "How have you been feeling since I saw you last?" Study staff will also review the previous AE form and inquire whether any of those events are continuing. Study clinicians will follow all AEs, regardless of severity, until resolution or until four weeks following completion of the trial. Each new or unresolved AE will be recorded on the AE case report form according to standard procedures. All AEs will be assigned a severity (mild, moderate, severe or life-threatening), as defined by the DAIDS Table for Grading Severity of Adult Adverse Experiences for HIV Prevention Trials (version 2.0).[128] The study clinician will review the information and offer an educated opinion about the relatedness of the event to the study drug. These data will be reviewed by the PI, the sponsor-site's medical director (Dr. Coffin), and by the study clinician on a biweekly basis.

A summary report of all AEs (including SAEs) will be prepared at least every six months (frequency determined by our IRB, DSMB, and the NIH), to be submitted to the DSMB, IRB and the NIH.

B. Serious Adverse Events (SAEs)

SAEs are defined as any fatal event, any immediately life-threatening event, any permanent or substantially disabling event, any event that requires or prolongs inpatient hospitalization, or any congenital anomaly. This category also includes any other important medical event that a study investigator judges to be serious because it may jeopardize the subject or require intervention to prevent one of the above reportable outcomes, or which would suggest a significant hazard, contraindication, side effect, or precaution. An unexpected event is one that is not described with respect to nature, severity, or frequency in the current protocol, investigator's brochure, or product labeling. All AEs that are both serious and unexpected will be reported to the DSMB and UCSF IRB, in writing, within 5 working days. If the SAE is fatal or life threatening, the PI will notify the FDA by telephone within 24 hours, with a follow-up written report within two working days.

As required, expedited (within 72 hours of knowledge of occurrence) reporting of SAEs to the NIDA will adhere to the following guidelines:

a. apply regardless of the investigator's assessment of the relatedness of the SAE to the intervention under study;

b. apply equally to trials requiring an Investigational New Drug (IND) application and those not requiring an IND application;

c. apply to any SAEs that occur during the post-treatment observation period defined by the protocol; and

d. apply to suicidal or homicidal behavior that causes an SAE in the participant or someone else (e.g., hospitalization or death).

SAE reporting will include a narrative that will provide details of relevant enrollment measures, medical history and physical findings, treatment compliance, participant reports of SAEs, and any other required information. The completed SAE report will contain: subject's study ID number, gender, age, the title and date of the SAE, and narrative explanation. The SAE form will track how the research staff was notified of the event, dates of consent, randomization, study enrollment for inclusion/exclusion, treatment received, outcome of study treatment, dates and circumstances of the hospitalization/death, whether alcohol or drugs were known to be involved, and participant status at last clinical or research contact. In cases of participant death, the report will also include appropriate substantiation from clinic records, and, whenever possible, copies of the death certificate, autopsy report, or medical record. The study site medical director, Dr. Coffin, will state whether the event was expected and assess its relatedness to the study medication or intervention.

If it becomes clear that the trial puts undue safety risk on study participants consideration will be given to stopping the trial after consultation with the DSMB, IRB and NIH Project Officer.

6.7.3 Venipuncture Risks

As part of study procedures, participants will be required to have their blood drawn four times over the course of their time in the study for safety lab assessments. Having blood drawn could result in pain, bruising around the needle site, risk of infection at the needle site and occasional failure in the vacuum tubes.

To protect against these risks all research study staff are trained phlebotomists.

6.7.4 Risk to Privacy and Confidentiality

Participating in the study presents a risk to the subject of loss of privacy and confidentiality, including unauthorized disclosure of confidential information; discomfort or embarrassment related to dealing with personal habits and lifestyle, including drug or alcohol use, and possible unwanted encounters with friends or associates.

To minimize the risk of loss of privacy and confidentiality, the consent form will inform participants of confidentiality guidelines. The study will follow HIPAA regulations for the protection of private health information for individuals. All study participants will sign the HIPAA Authorization Form. A Certificate of Confidentiality has been obtained from NIDA to offer protection for the privacy of subjects. With this certificate, the researcher cannot be forced to disclose information that may identify them, even by a court subpoena, in any

federal, state or local civil, criminal, administrative, legislative or other proceeding. Exceptions to confidentiality for research participants are those required by law and include suspicion of child abuse, elder abuse, and threat of imminent action on suicidal or homicidal behavior. Participants will be informed of these exceptions in the informed consent process.

Records that have personal identifiers will be stored in a locked cabinet separate from the research records, which will contain only the participant's assigned ID number. Only the research team will have access to non-anonymous records. All research data are maintained in binders in locked cabinets. Consent form, which contain names, are stored separately.

6.7.5 Electrocardiogram (ECG) Risks

Participating in the study presents minor risks to the subject involved in using an ECG machine. Those risks include the patch adhesives being cold and sticky and they may develop a rash that often goes away without treatment.

All participants will be informed of these risks during the consent process.

6.7.6 Randomization Risks

Subjects are assigned to a treatment arm by a computer-generated randomization code with a 2:1 allocation ratio for lorcaserin and placebo. Assignment of two-thirds of participants to the lorcaserin arm was specifically chosen to improve our ability to detect adverse events in the active treatment arm, while preserving our ability to assess recruitment in a study in which randomization to placebo is likely. To minimize any risk from randomization, study staff will explain to potential participants during the consent process what being randomized means. In the informed consent, subjects will be told,

> "**Be randomized:** When you are randomized, a computer will randomly assign you to either the lorcaserin or placebo group. Neither you nor your study doctor, nurse, or counselor can choose the group you will be in. This is a double-blind study, which means neither you nor your study doctor, nurse, or counselor will know which group you are in. You will have a 2:1 chance of being in the lorcaserin or placebo group.
>
> - If you are in the Lorcaserin group, you will receive lorcaserin for 12 weeks.
> - If you are in the placebo group, you will receive placebo (an inactive substance) for 12 weeks."

6.7.7 Adverse Event Reporting

A. Each adverse event (AE) will be classified by the study clinician as serious or non-serious, and appropriate reporting procedures will be followed; these decisions will be reviewed on a real-time basis by the study clinicians. An AE is defined as any reaction, side effect, or untoward event that occurs during the course of the clinical trial, whether or not the event is considered medication-related. A new illness, symptom, or worsening of a pre-existing condition or abnormality is considered an AE. Stable chronic conditions that are present prior to clinical trial

entry and do not worsen are not considered AEs. For this study, AEs will include symptoms reported by the patient and abnormal measures of clinical importance noted by study staff.

Study staff will assess participants for any medical or psychiatric side effects by asking the participant, “How have you been feeling since I saw you last?” Study staff will also review the previous AE form and inquire whether any of those events are continuing. Study clinicians will follow all AEs, regardless of severity, until resolution or until four weeks following completion of the trial by calling and checking in with the participant. Each new or unresolved AE will be recorded on the AE case report form according to standard procedures. All AEs will be assigned a severity (mild, moderate, severe or life-threatening), as defined by the DAIDS Table for Grading Severity of Adult Adverse Experiences for HIV Prevention Trials (version 2.0).[128] The study clinician will review the information and offer an educated opinion about the relatedness of the event to the study drug. These data will be reviewed by the PI, the sponsor-site’s medical director (Dr. Coffin), and by the study clinician on a biweekly basis.

A summary report of all AEs (including SAEs) will be prepared at least every six months (frequency determined by our IRB, DSMB, and the NIH), to be submitted to the DSMB, IRB and the NIH.

6.8 Clinical Trial Registration

This investigation has been registered on Clinicaltrials.gov. Its registration number is: NCT03192995.

Please see the signed and dated Form FDA 3674 in Appendix 8.

7.0 CHEMISTRY, MANUFACTURING AND CONTROL INFORMATION

7.1 Chemistry

Please see the package insert from BELVIQ® XR in Appendix 1.

7.2 Manufacture

7.2.1 Purity:

Please see Appendix 2a and 2b for Certificate of Analysis and Bovine Spongiform Encephalopathy Free (BSE/TSE) Certificate.

7.2.2 Investigational Drug Label

[redacted]

7.2.3 Claim for Categorical Exclusion for Environmental Assessment

Under the provisions of 21 CFR 25.31, the investigator requests that exclusion be granted from the requirements for environmental assessment. The investigational new drug is intended for use in clinical studies on research in which waste will be controlled. In addition, the amount of waste expected to enter the environment may reasonably be expected to be nontoxic.

8.0 PHARMACOLOGY AND TOXICOLOGY INFORMATION

Please see BELVIQ® XR package insert in Appendix 1.

9.0 PREVIOUS HUMAN EXPERIENCE

Please see Appendices 3a-3c for articles containing human research done involving lorcaserin.

10.0 ADDITIONAL INFORMATION

None

11.0 RELEVANT INFORMATION

None

12.0 APPENDICES COVER SHEET

12.1 Appendix 1: Reference for Section 5.0, Package Insert and Section 7.0, Chemistry, Manufacturing, and Control Information

App 1: BELVIQ® XR Package Insert

12.2 Appendix 2a and 2b: Reference for Section 7.0, Chemistry, Manufacturing and Control Information

App 2a: Certificate of Analysis
App 2b: Bovine Spongiform Encephalopathy Free (BSE/TSE) Certificate

12.3 Appendix 3a-3c: Reference for Section 9.0, Previous Human Experience

App 3a: Fidler 2011 BLOSSOM Trial
App 3b: O'Neil 2012 BLOOM-DM Study
App 3c: Howell 2016 Pharmacological Review

12.4 Appendix 4: FDA Form 1572

12.5 Appendix 5: PI's CV

12.6 Appendix 6: Sub-Investigator's CV

12.7 Appendix 7: Form FDA 3454

12.8 Appendix 8: Form FDA 3674

12.1 Appendix 1: Cover Sheet

BELVIQ® XR Package Insert

12.2 Appendix 2a and 2b: Cover Sheet

App 2a:	Certificate of Analysis
App 2b:	Bovine Spongiform Encephalopathy Free (BSE/TSE) Certificate

12.3 Appendix 3a-3c: Cover Sheet

App 3a:	Fidler 2011 BLOSSOM Trial
App 3b:	O’Neil 2012 BLOOM-DM Study
App 3c:	Howell 2016 Pharmacological Review

12.4 Appendix 4: Cover Sheet

FDA Form 1572

12.5 Appendix 5: Cover Sheet

PI's CV

12.6 Appendix 6: Cover Sheet

Sub-Investigator's CV

12.7 Appendix 7: Cover Sheet

Form FDA 3454

12.8 Appendix 8: Cover Sheet

Form FDA 3674

References

1. Arena Pharmaceuticals. *Belviq XR (lorcaserin HCl) Prescribing Information* [Package Insert] 2012 August 1, 2012 [cited 2014 April 16, 2014]; Available from: http://www.Belviq XR.com/documents/Belviq XR_Prescribing_information.pdf.
2. Levin, E.D., et al., *Lorcaserin, a 5-HT2C agonist, decreases nicotine self-administration in female rats.* J Pharmacol Exp Ther, 2011. **338**(3): p. 890-6.
3. Higgins, G.A., et al., *The 5-HT2C receptor agonist lorcaserin reduces nicotine self-administration, discrimination, and reinstatement: relationship to feeding behavior and impulse control.* Neuropsychopharmacology, 2012. **37**(5): p. 1177-91.
4. Volkow, N.D. and R.A. Wise, *How can drug addiction help us understand obesity?* Nat Neurosci, 2005. **8**(5): p. 555-60.
5. Kenny, P.J., *Common cellular and molecular mechanisms in obesity and drug addiction.* Nat Rev Neurosci, 2011. **12**(11): p. 638-51.
6. Volkow, N.D. and C.P. O'Brien, *Issues for DSM-V: should obesity be included as a brain disorder?* Am J Psychiatry, 2007. **164**(5): p. 708-10.
7. Higgins, G.A., E.M. Sellers, and P.J. Fletcher, *From obesity to substance abuse: therapeutic opportunities for 5-HT2C receptor agonists.* Trends Pharmacol Sci, 2013. **34**(10): p. 560-70.
8. Higgins, G.A., et al., *Evaluation of chemically diverse 5-HT(2)c receptor agonists on behaviours motivated by food and nicotine and on side effect profiles.* Psychopharmacology (Berl), 2013. **226**(3): p. 475-90.
9. Fletcher, P.J., et al., *Impulsive action induced by amphetamine, cocaine and MK801 is reduced by 5-HT(2C) receptor stimulation and 5-HT(2A) receptor blockade.* Neuropharmacology, 2011. **61**(3): p. 468-77.
10. Navarra, R., et al., *The 5-HT(2C) receptor agonist WAY-163909 decreases impulsivity in the 5-choice serial reaction time test.* Behav Brain Res, 2008. **188**(2): p. 412-5.
11. Chan, E.W., et al., *Efficacy and safety of lorcaserin in obese adults: a meta-analysis of 1-year randomized controlled trials (RCTs) and narrative review on short-term RCTs.* Obes Rev, 2013. **14**(5): p. 383-92.
12. Smith, S.R., et al., *Multicenter, placebo-controlled trial of lorcaserin for weight management.* N Engl J Med, 2010. **363**(3): p. 245-56.
13. Fidler, M.C., et al., *A one-year randomized trial of lorcaserin for weight loss in obese and overweight adults: the BLOSSOM trial.* J Clin Endocrinol Metab, 2011. **96**(10): p. 3067-77.
14. O'Neil, P.M., et al., *Randomized placebo-controlled clinical trial of lorcaserin for weight loss in type 2 diabetes mellitus: the BLOOM-DM study.* Obesity (Silver Spring), 2012. **20**(7): p. 1426-36.
15. Shram, M.J., et al., *Evaluation of the abuse potential of lorcaserin, a serotonin 2C (5-HT2C) receptor agonist, in recreational polydrug users.* Clin Pharmacol Ther, 2011. **89**(5): p. 683-92.
16. Riezzo, I., et al., *Side effects of cocaine abuse: multiorgan toxicity and pathological consequences.* Curr Med Chem, 2012. **19**(33): p. 5624-46.
17. Karila, L., et al., *Diagnosis and consequences of cocaine addiction.* Curr Med Chem, 2012. **19**(33): p. 5612-8.
18. Substance Abuse and Mental Health Services Administration, *Drug Abuse Warning Network, 2011: National Estimates of Drug-Related Emergency Department Visits* in *DAWN*, SAMHSA, Editor. 2013, HHS: Rockville, MD.
19. United Nations Office on Drugs and Crime, *World Drug Report 2009*, UNODC, Editor. 2009: Vienna, Austria.
20. Degenhardt, L., et al., *Toward a global view of alcohol, tobacco, cannabis, and cocaine use: findings from the WHO World Mental Health Surveys.* PLoS Med, 2008. **5**(7): p. e141.

21. Anthony, J., L. Warner, and R. Kessler, *Comparative epidemiology of dependence on tobacco, alcohol, controlled substances, and inhalants: Basic findings from the National Comorbidity Survey.* Experimental and Clinical Psychopharmacology, 1994. **23**: p. 244.
22. Degenhardt, L., et al., *The global epidemiology and burden of psychostimulant dependence: Findings from the Global Burden of Disease Study 2010.* Drug Alcohol Depend, 2014.
23. Vandhuick, O., et al., *[Drug addiction and cardiovascular pathologies].* J Mal Vasc, 2004. **29**(5): p. 243-8.
24. Kloner, R.A., et al., *The effects of acute and chronic cocaine use on the heart.* Circulation, 1992. **85**(2): p. 407-19.
25. McCord, J., et al., *Management of cocaine-associated chest pain and myocardial infarction: a scientific statement from the American Heart Association Acute Cardiac Care Committee of the Council on Clinical Cardiology.* Circulation, 2008. **117**(14): p. 1897-907.
26. Phillips, K., et al., *Cocaine cardiotoxicity: a review of the pathophysiology, pathology, and treatment options.* Am J Cardiovasc Drugs, 2009. **9**(3): p. 177-96.
27. Steinhauer, J.R. and J.B. Caulfield, *Spontaneous coronary artery dissection associated with cocaine use: a case report and brief review.* Cardiovasc Pathol, 2001. **10**(3): p. 141-5.
28. Sanchez, T., et al., *Human immunodeficiency virus (HIV) risk, prevention, and testing behaviors--United States, National HIV Behavioral Surveillance System: men who have sex with men, November 2003-April 2005.* MMWR Surveill Summ, 2006. **55**(6): p. 1-16.
29. SAMHSA, *National Survey on Drug Use and Health.* Office of Applied Studies, 2004.
30. Raymond, H.F., *Estimated number of stimulant using men-who-have-sex-with-men in San Francisco*, G.M. Santos, Editor. 2012: San Francisco, CA.
31. Raymond, H.F., *National HIV Behavioral Surveillance MSM cycle 4 stimulant use data* G.M. Santos, Editor. 2014: San Francisco, CA.
32. Raymond, H.F., et al., *A new trend in the HIV epidemic among men who have sex with men, San Francisco, 2004-2011.* J Acquir Immune Defic Syndr, 2013. **62**(5): p. 584-9.
33. San Francisco Department of Public Health, *2012 HIV/AIDS Epidemiology Annual Report*, H.E. Section, Editor. 2013, San Francisco Department of Public Health San Francisco
34. San Francisco Department of Public Health, *2013 HIV/AIDS Epidemiology Annual Report*, H.E. Section, Editor. 2014, San Francisco Department of Public Health San Francisco
35. Prejean, J., et al., *Estimated HIV incidence in the United States, 2006-2009.* PLoS One, 2011. **6**(8): p. e17502.
36. Hall, H.I., et al., *Estimation of HIV incidence in the United States.* Jama, 2008. **300**(5): p. 520-9.
37. Purcell, D., et al., *Calculating HIV and Syphilis Rates for Risk Groups: Estimating the National Population Size of Men Who Have Sex with Men*, in *National STD Prevention Conference*. 2010: Atlanta, GA
38. San Francisco Department of Public Health, *2010 HIV/AIDS Epidemiology Annual Report*, H.E. Section, Editor. 2011, San Francisco Department of Public Health San Francisco
39. ONAP, *National HIV/AIDS Strategy for the United States*, T.W.H.O.o.N.A. Policy, Editor. 2010: Washington, DC.
40. Volkow, N.D., et al., *Stimulant-induced enhanced sexual desire as a potential contributing factor in HIV transmission.* Am J Psychiatry, 2007. **164**(1): p. 157-60.
41. Colfax, G., et al., *Longitudinal patterns of methamphetamine, popper (amyl nitrite), and cocaine use and high-risk sexual behavior among a cohort of san francisco men who have sex with men.* J Urban Health, 2005. **82**(1 Suppl 1): p. i62-70.
42. Shoptaw, S., et al., *Stimulant abuse treatment as HIV prevention.* J Addict Dis, 1998. **17**(4): p. 19-32.

43. Celentano, D.D., et al., *Associations between substance use and sexual risk among very young men who have sex with men.* Sex Transm Dis, 2006. **33**(4): p. 265-71.
44. Melis, M.R. and A. Argiolas, *Dopamine and sexual behavior.* Neurosci Biobehav Rev, 1995. **19**(1): p. 19-38.
45. Cunningham, K.A. and N.C. Anastasio, *Serotonin at the nexus of impulsivity and cue reactivity in cocaine addiction.* Neuropharmacology, 2014. **76 Pt B**: p. 460-78.
46. Winhusen, T., et al., *Impulsivity is associated with treatment non-completion in cocaine- and methamphetamine-dependent patients but differs in nature as a function of stimulant-dependence diagnosis.* J Subst Abuse Treat, 2013. **44**(5): p. 541-7.
47. McKirnan, D.J., D.G. Ostrow, and B. Hope, *Sex, drugs and escape: a psychological model of HIV-risk sexual behaviours.* AIDS Care, 1996. **8**(6): p. 655-69.
48. Lejuez, C.W., et al., *Differences in impulsivity and sexual risk behavior among inner-city crack/cocaine users and heroin users.* Drug Alcohol Depend, 2005. **77**(2): p. 169-75.
49. Parsons, J.T. and P.N. Halkitis, *Sexual and drug-using practices of HIV-positive men who frequent public and commercial sex environments.* AIDS Care, 2002. **14**(6): p. 815-26.
50. Purcell, D.W., et al., *Substance use and sexual transmission risk behavior of HIV-positive men who have sex with men.* J Subst Abuse, 2001. **13**(1-2): p. 185-200.
51. Mansergh, G., et al., *The Circuit Party Men's Health Survey: findings and implications for gay and bisexual men.* Am J Public Health, 2001. **91**(6): p. 953-8.
52. Mimiaga, M.J., et al., *Walking the line: stimulant use during sex and HIV risk behavior among Black urban MSM.* Drug Alcohol Depend, 2010. **110**(1-2): p. 30-7.
53. Colfax, G., et al., *Substance use and sexual risk: a participant- and episode-level analysis among a cohort of men who have sex with men.* Am J Epidemiol, 2004. **159**(10): p. 1002-12.
54. Ober, A., et al., *Factors associated with event-level stimulant use during sex in a sample of older, low-income men who have sex with men in Los Angeles.* Drug Alcohol Depend, 2009. **102**(1-3): p. 123-9.
55. Mackesy-Amiti, M.E., M. Fendrich, and T.P. Johnson, *Symptoms of substance dependence and risky sexual behavior in a probability sample of HIV-negative men who have sex with men in Chicago.* Drug Alcohol Depend, 2010. **110**(1-2): p. 38-43.
56. Lambert, G., et al., *Correlates of unprotected anal sex at last sexual episode: analysis from a surveillance study of men who have sex with men in Montreal.* AIDS Behav, 2011. **15**(3): p. 584-95.
57. Bowers, J.R., et al., *Predictors of HIV Sexual Risk Behavior among Men Who Have Sex with Men, Men Who Have Sex with Men and Women, and Transgender Women.* Int J Sex Health, 2012. **24**(4): p. 290-302.
58. Santos, G.M., et al., *Dose-response associations between number and frequency of substance use and high-risk sexual behaviors among HIV-negative substance-using men who have sex with men (SUMSM) in San Francisco.* J Acquir Immune Defic Syndr, 2013. **63**(4): p. 540-4.
59. Ostrow, D.G., et al., *Specific sex drug combinations contribute to the majority of recent HIV seroconversions among MSM in the MACS.* J Acquir Immune Defic Syndr, 2009. **51**(3): p. 349-55.
60. Koblin, B.A., et al., *Risk factors for HIV infection among men who have sex with men.* AIDS, 2006. **20**(5): p. 731-9.
61. Ostrow, D.G., et al., *A case-control study of human immunodeficiency virus type 1 seroconversion and risk-related behaviors in the Chicago MACS/CCS Cohort, 1984-1992. Multicenter AIDS Cohort Study. Coping and Change Study.* Am J Epidemiol, 1995. **142**(8): p. 875-83.
62. Carrico, A.W., et al., *Psychological processes and stimulant use among men who have sex with men.* Drug Alcohol Depend, 2012. **123**(1-3): p. 79-83.

63. Hinkin, C.H., et al., *Drug use and medication adherence among HIV-1 infected individuals.* AIDS Behav, 2007. **11**(2): p. 185-94.
64. Carrico, A.W., *Substance use and HIV disease progression in the HAART era: implications for the primary prevention of HIV.* Life Sci, 2011. **88**(21-22): p. 940-7.
65. Shoptaw, S., et al., *Cumulative exposure to stimulants and immune function outcomes among HIV-positive and HIV-negative men in the Multicenter AIDS Cohort Study.* Int J STD AIDS, 2012. **23**(8): p. 576-80.
66. Cook, J.A., et al., *Crack cocaine, disease progression, and mortality in a multicenter cohort of HIV-1 positive women.* AIDS, 2008. **22**(11): p. 1355-63.
67. Carrico, A.W., et al., *Stimulant use is associated with immune activation and depleted tryptophan among HIV-positive persons on anti-retroviral therapy.* Brain Behav Immun, 2008. **22**(8): p. 1257-62.
68. Roth, M.D., et al., *Cocaine enhances human immunodeficiency virus replication in a model of severe combined immunodeficient mice implanted with human peripheral blood leukocytes.* J Infect Dis, 2002. **185**(5): p. 701-5.
69. Bagasra, O. and R.J. Pomerantz, *Human immunodeficiency virus type 1 replication in peripheral blood mononuclear cells in the presence of cocaine.* J Infect Dis, 1993. **168**(5): p. 1157-64.
70. Cohen, M.S., et al., *Prevention of HIV-1 infection with early antiretroviral therapy.* N Engl J Med, 2011. **365**(6): p. 493-505.
71. Santos, G.M., M. Das, and G.N. Colfax, *Interventions for non-injection substance use among US men who have sex with men: what is needed.* AIDS Behav, 2011. **15 Suppl 1**: p. S51-6.
72. Mackesy-Amiti, M.E., M. Fendrich, and T.P. Johnson, *Substance-related problems and treatment among men who have sex with men in comparison to other men in Chicago.* J Subst Abuse Treat, 2009. **36**(2): p. 227-33.
73. Shallow, S., *Persons attending substance abuse treatment programs.* 2004, San Francisco Department of Public Health: San Francisco, CA.
74. Santos, G.M., et al., *Substance use and drinking outcomes in Personalized Cognitive Counseling randomized trial for episodic substance-using men who have sex with men.* Drug Alcohol Depend, 2014. **138**: p. 234-9.
75. Coffin, P.O., et al., *Adapted Personalized Cognitive Counseling for Episodic Substance-Using Men Who Have Sex with Men: A Randomized Controlled Trial.* AIDS Behav, 2014.
76. Dutra, L., et al., *A meta-analytic review of psychosocial interventions for substance use disorders.* Am J Psychiatry, 2008. **165**(2): p. 179-87.
77. Kampman, K.M., *The search for medications to treat stimulant dependence.* Addict Sci Clin Pract, 2008. **4**(2): p. 28-35.
78. Alvarez, Y., et al., *Antipsychotic drugs in cocaine dependence: a systematic review and meta-analysis.* J Subst Abuse Treat, 2013. **45**(1): p. 1-10.
79. Jorenby, D.E., et al., *A controlled trial of sustained-release bupropion, a nicotine patch, or both for smoking cessation.* N Engl J Med, 1999. **340**(9): p. 685-91.
80. Fiellin, D.A., *Substance use and disorders in HIV-infected patients: Impact and new treatment strategies.* Top HIV Med, 2004. **12**(3): p. 77-82.
81. Johnson, R.E., et al., *A comparison of levomethadyl acetate, buprenorphine, and methadone for opioid dependence.* N Engl J Med, 2000. **343**(18): p. 1290-7.
82. Colfax, G.N., et al., *Mirtazapine to reduce methamphetamine use: a randomized controlled trial.* Arch Gen Psychiatry, 2011. **68**(11): p. 1168-75.
83. Rothman, R.B., B.E. Blough, and M.H. Baumann, *Dopamine/serotonin releasers as medications for stimulant addictions.* Prog Brain Res, 2008. **172**: p. 385-406.

84. Alex, K.D. and E.A. Pehek, *Pharmacologic mechanisms of serotonergic regulation of dopamine neurotransmission.* Pharmacol Ther, 2007. **113**(2): p. 296-320.
85. Di Matteo, V., et al., *SB 242084, a selective serotonin2C receptor antagonist, increases dopaminergic transmission in the mesolimbic system.* Neuropharmacology, 1999. **38**(8): p. 1195-205.
86. Anastasio, N.C., et al., *Variation within the serotonin (5-HT) 5-HT2C receptor system aligns with vulnerability to cocaine cue reactivity.* Transl Psychiatry, 2014. **4**: p. e369.
87. Anastasio, N.C., et al., *Functional status of the serotonin 5-HT2C receptor (5-HT2CR) drives interlocked phenotypes that precipitate relapse-like behaviors in cocaine dependence.* Neuropsychopharmacology, 2014. **39**(2): p. 370-82.
88. Bubar, M.J. and K.A. Cunningham, *Serotonin 5-HT2A and 5-HT2C receptors as potential targets for modulation of psychostimulant use and dependence.* Curr Top Med Chem, 2006. **6**(18): p. 1971-85.
89. Bubar, M.J. and K.A. Cunningham, *Prospects for serotonin 5-HT2R pharmacotherapy in psychostimulant abuse.* Prog Brain Res, 2008. **172**: p. 319-46.
90. Burton, C.L., et al., *Antagonizing 5-HT(2)A receptors with M100907 and stimulating 5-HT(2)C receptors with Ro60-0175 blocks cocaine-induced locomotion and zif268 mRNA expression in Sprague-Dawley rats.* Behav Brain Res, 2013. **240**: p. 171-81.
91. Craige, C.P. and E.M. Unterwald, *Serotonin (2C) receptor regulation of cocaine-induced conditioned place preference and locomotor sensitization.* Behav Brain Res, 2013. **238**: p. 206-10.
92. Cunningham, K.A., et al., *Selective serotonin 5-HT(2C) receptor activation suppresses the reinforcing efficacy of cocaine and sucrose but differentially affects the incentive-salience value of cocaine- vs. sucrose-associated cues.* Neuropharmacology, 2011. **61**(3): p. 513-23.
93. Filip, M., et al., *Behavioral evidence for the significance of serotoninergic (5-HT) receptors in cocaine addiction.* Addict Biol, 2010. **15**(3): p. 227-49.
94. Filip, M., et al., *Pharmacological and genetic interventions in serotonin (5-HT)(2C) receptors to alter drug abuse and dependence processes.* Brain Res, 2012. **1476**: p. 132-53.
95. Katsidoni, V., K. Apazoglou, and G. Panagis, *Role of serotonin 5-HT2A and 5-HT2C receptors on brain stimulation reward and the reward-facilitating effect of cocaine.* Psychopharmacology (Berl), 2011. **213**(2-3): p. 337-54.
96. Leggio, G.M., et al., *Serotonin2C receptors in the medial prefrontal cortex facilitate cocaine-induced dopamine release in the rat nucleus accumbens.* Neuropharmacology, 2009. **56**(2): p. 507-13.
97. Manvich, D.F., H.L. Kimmel, and L.L. Howell, *Effects of serotonin 2C receptor agonists on the behavioral and neurochemical effects of cocaine in squirrel monkeys.* J Pharmacol Exp Ther, 2012. **341**(2): p. 424-34.
98. Pockros, L.A., et al., *5-HT(2A) receptor blockade and 5-HT(2C) receptor activation interact to reduce cocaine hyperlocomotion and Fos protein expression in the caudate-putamen.* Synapse, 2012. **66**(12): p. 989-1001.
99. Thomsen, W.J., et al., *Lorcaserin, a novel selective human 5-hydroxytryptamine2C agonist: in vitro and in vivo pharmacological characterization.* J Pharmacol Exp Ther, 2008. **325**(2): p. 577-87.
100. Grottick, A.J., P.J. Fletcher, and G.A. Higgins, *Studies to investigate the role of 5-HT(2C) receptors on cocaine- and food-maintained behavior.* J Pharmacol Exp Ther, 2000. **295**(3): p. 1183-91.
101. Pelloux, Y., et al., *Reduced forebrain serotonin transmission is causally involved in the development of compulsive cocaine seeking in rats.* Neuropsychopharmacology, 2012. **37**(11): p. 2505-14.

102. Kohut, S., J. Bergman, and N. Mello, *Pretreatment with either a 5-HT2C agonist or 5-HT2A antagonist decreases cocaine self-administration.* The FASEB Journal, 2014. **28**(1, Supplement 848.9).

103. Cunningham, K.A., et al., *Synergism between a serotonin 5-HT2A receptor (5-HT2AR) antagonist and 5-HT2CR agonist suggests new pharmacotherapeutics for cocaine addiction.* ACS Chem Neurosci, 2013. **4**(1): p. 110-21.

104. Neisewander, J.L. and J.I. Acosta, *Stimulation of 5-HT2C receptors attenuates cue and cocaine-primed reinstatement of cocaine-seeking behavior in rats.* Behav Pharmacol, 2007. **18**(8): p. 791-800.

105. Fletcher, P.J., et al., *Characterizing the effects of 5-HT(2C) receptor ligands on motor activity and feeding behaviour in 5-HT(2C) receptor knockout mice.* Neuropharmacology, 2009. **57**(3): p. 259-67.

106. Fletcher, P.J., et al., *The 5-HT2C receptor agonist Ro60-0175 reduces cocaine self-administration and reinstatement induced by the stressor yohimbine, and contextual cues.* Neuropsychopharmacology, 2008. **33**(6): p. 1402-12.

107. Burbassi, S. and L. Cervo, *Stimulation of serotonin2C receptors influences cocaine-seeking behavior in response to drug-associated stimuli in rats.* Psychopharmacology (Berl), 2008. **196**(1): p. 15-27.

108. Zou, G., *A modified poisson regression approach to prospective studies with binary data.* Am J Epidemiol, 2004. **159**(7): p. 702-6.

109. Hunt, M.K., et al., *Construct validity of the Balloon Analog Risk Task (BART): associations with psychopathy and impulsivity.* Assessment, 2005. **12**(4): p. 416-28.

110. Wingrove, J. and A.J. Bond, *Impulsivity: A State as Well as Trait Variable. Does Mood Awareness Explain Low Correlations Between Trait and Behavioral Measures of Impulsivity.* Person. Individ. Diff., 1997. **22**(3): p. 333-339.

111. Bancroft, J. and Z. Vukadinovic, *Sexual addiction, sexual compulsivity, sexual impulsivity, or what? Toward a theoretical model.* J Sex Res, 2004. **41**(3): p. 225-34.

112. Tziortzis, D., et al., *The relationship between impulsivity and craving in cocaine- and methamphetamine-dependent volunteers.* Pharmacol Biochem Behav, 2011. **98**(2): p. 196-202.

113. Halikas, J.A., et al., *The measurement of craving in cocaine patients using the Minnesota Cocaine Craving Scale.* Compr Psychiatry, 1991. **32**(1): p. 22-7.

114. Gossop, M., et al., *The Severity of Dependence Scale (SDS): psychometric properties of the SDS in English and Australian samples of heroin, cocaine and amphetamine users.* Addiction, 1995. **90**(5): p. 607-14.

115. Cole, S.R. and M.A. Hernan, *Fallibility in estimating direct effects.* Int J Epidemiol, 2002. **31**(1): p. 163-5.

116. McCann, D.J. and S.H. Li, *A Novel, Nonbinary Evaluation of Success and Failure Reveals Bupropion Efficacy Versus Methamphetamine Dependence: Reanalysis of a Multisite Trial.* CNS Neurosci Ther, 2011.

117. Fals-Stewart, W., et al., *The timeline followback reports of psychoactive substance use by drug-abusing patients: psychometric properties.* J Consult Clin Psychol, 2000. **68**(1): p. 134-44.

118. Kranzler, H.R., et al., *Targeted naltrexone for problem drinkers.* J Clin Psychopharmacol, 2009. **29**(4): p. 350-7.

119. Turner, C., et al., *Race/ethnicity and education predict engagement in text messaging: Digital divide in ecological momentary assessments among substance-using MSM in San Francisco*, in *University of California San Francisco Health Disparities Research Symposium IX*, U.o.C.S. Francisco, Editor. 2015: San Francisco, CA.

120. PharmChem Inc. *Drugs of Abuse Sweat Patch*. 2013 2013 [cited 2014 12/20/14]; Available from: http://www.pharmchem.com/default.asp?page=2.25.
121. Spiehler, V., et al., *Enzyme immunoassay validation for qualitative detection of cocaine in sweat.* Clin Chem, 1996. **42**(1): p. 34-8.
122. Preston, K.L., et al., *Monitoring cocaine use in substance-abuse-treatment patients by sweat and urine testing.* J Anal Toxicol, 1999. **23**(5): p. 313-22.
123. Macalino, G.E., et al., *Risk behaviors by audio computer-assisted self-interviews among HIV-seropositive and HIV-seronegative injection drug users.* AIDS Educ Prev, 2002. **14**(5): p. 367-78.
124. Koblin, B., et al., *Effects of a behavioural intervention to reduce acquisition of HIV infection among men who have sex with men: the EXPLORE randomised controlled study.* Lancet, 2004. **364**(9428): p. 41-50.
125. Halpern, B. and A. Halpern, *Safety assessment of FDA-approved (orlistat and lorcaserin) anti-obesity medications.* Expert Opin Drug Saf, 2015. **14**(2): p. 305-15.
126. Frank, C., *Recognition and treatment of serotonin syndrome.* Can Fam Physician, 2008. **54**(7): p. 988-92.
127. Dunkley, E.J., et al., *The Hunter Serotonin Toxicity Criteria: simple and accurate diagnostic decision rules for serotonin toxicity.* QJM, 2003. **96**(9): p. 635-42.
128. U.S. Department of Health and Human Services, N.I.o.H., National Institute of Allergy and Infectious Diseases, Division of AIDS, , *Table for Grading the Severity of Adult and Pediatric Adverse Events, Version 2.0*. 2014.
